# Functional Convergence of Autonomic and Sensorimotor Processing in the Lateral Cerebellum

**DOI:** 10.1016/j.celrep.2020.107867

**Published:** 2020-07-07

**Authors:** Vincenzo Romano, Aoibhinn L. Reddington, Silvia Cazzanelli, Roberta Mazza, Yang Ma, Christos Strydis, Mario Negrello, Laurens W.J. Bosman, Chris I. De Zeeuw

**Affiliations:** 1 Department of Neuroscience, Erasmus MC, 3015 GE Rotterdam, the Netherlands; 2 Netherlands Institute for Neuroscience, Royal Academy of Arts and Sciences, 1105 BA Amsterdam, the Netherlands

**Keywords:** cerebellum, Purkinje cells, sensorimotor behavior, respiration, whisking, whisker stimulation, phase dependent responses, electrophysiology, optogenetics, plasticity

## Abstract

The cerebellum is involved in the control of voluntary and autonomic rhythmic behaviors, yet it is unclear to what extent it coordinates these in concert. We studied Purkinje cell activity during unperturbed and perturbed respiration in lobules simplex, crus 1, and crus 2. During unperturbed (eupneic) respiration, complex spike and simple spike activity encode the phase of ongoing sensorimotor processing. In contrast, when the respiratory cycle is perturbed by whisker stimulation, mice concomitantly protract their whiskers and advance their inspiration in a phase-dependent manner, preceded by increased simple spike activity. This phase advancement of respiration in response to whisker stimulation can be mimicked by optogenetic stimulation of Purkinje cells and prevented by cell-specific genetic modification of their AMPA receptors, hampering increased simple spike firing. Thus, the impact of Purkinje cell activity on respiratory control is context and phase dependent, highlighting a coordinating role for the cerebellar hemispheres in aligning autonomic and sensorimotor behaviors.

## Highlights

• During unperturbed respiration, Purkinje cells signal ongoing sensorimotor processing
• After perturbation, mice advance their simple spike activity, whisking, and inspiration
• Altering simple spike activity affects the impact of whisker stimulation on respiration
• Cerebellar coordination of autonomic and sensorimotor behaviors is context dependent

## Graphical Abstract

**Figure:**
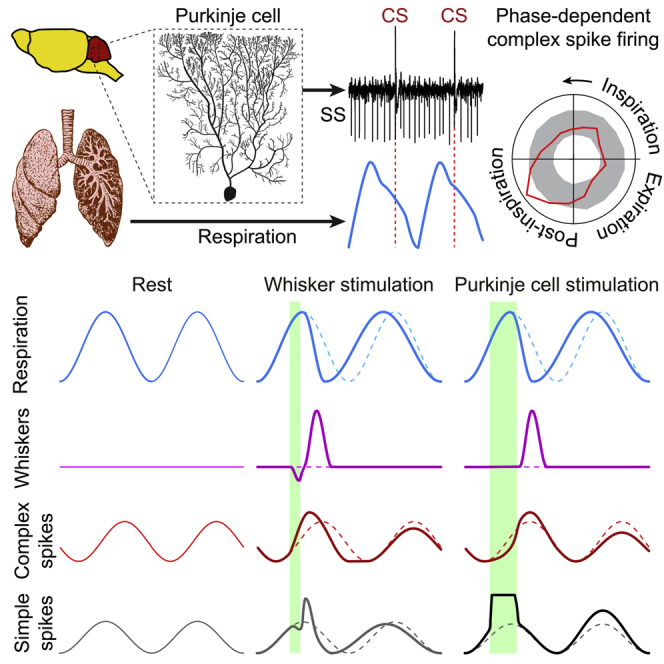

## Teaser

Romano et al. show that cerebellar Purkinje cell activity follows the respiratory rhythm during rest. Triggered by sensory input, Purkinje cells can alter their activity and thereby accelerate the timing of the next inspiration. Concomitantly, they also augment whisker movements, highlighting a coordinating role in aligning autonomic and sensorimotor behaviors.

## Introduction

Rhythmic behaviors are part of the everyday life of mammals. They can emerge from predominantly conscious activities, such as locomotion, licking, or whisking, but also from more subconscious behaviors, such as heartbeat or respiration. Speed, amplitude, and phase of rhythmic movements depend on the behavioral demands and context, and thereby they depend on each other (Cao et al., 2012; Kurnikova et al., 2017; Moore et al., 2013; Welker, 1964). Accordingly, many of the motor domains involved in rhythmic movements serve multiple functions, and many of these can be coordinated in a concerted action. For example, inspiration is driven by the diaphragm and intercostal muscles, which are also involved in postural control (Hodges and Gandevia, 2000; Rimmer et al., 1995), and respiration and posture are synergistically controlled during processes such as vocalization, swimming, or parturition (Holstege, 2014; Jakovljevic and McConnell, 2009; Tomori and Widdicombe, 1969).

When different forms of sensorimotor behaviors have to be coordinated, the olivocerebellar system is often involved in optimal fine-tuning in time and space (Kitazawa et al., 1998; Owens et al., 2018; Vinueza Veloz et al., 2015). This presumably not only holds for non-rhythmic behaviors, but also for rhythmic behaviors like respiration (Cao et al., 2012; Critchley et al., 2015; Gozal et al., 1995; Isaev et al., 2002; Liu et al., 2020; McKay et al., 2003; Park et al., 2016; Parsons et al., 2001; Raux et al., 2013). Accordingly, rare, but dramatic, cases of sudden infant death syndrome (SIDS) have been attributed to acute respiratory arrest in relation to inferior olivary hypoplasia or delayed maturation of the cerebellar cortex (Cortez and Kinney, 1996; Cruz-Sánchez et al., 1997; Harper, 2000; Katsetos et al., 2014; Lavezzi et al., 2013), while cerebellar dysfunction has been observed in congenital central hypoventilation syndrome, which entails the inability to react to dyspnea (Harper et al., 2005, 2015; Kumar et al., 2008). Likewise, patients with a cerebellar tumor or hemorrhage frequently need mechanical ventilation after neurosurgery, often showing a relatively slow recovery of respiration (Arnone et al., 2017; Chen et al., 2005; Gewaltig and Diesmann, 2007; Lee et al., 2013; Tsitsopoulos et al., 2012). Moreover, most cerebellar ataxia patients have trouble modulating their breathing during exercise (De Joanna et al., 2008; Deger et al., 1999; Ebert et al., 1995). Thus, there is ample evidence for a role of the olivocerebellar system in controlling respiration and adjusting it according to behavioral demands, pointing toward a role in synergistic integration of autonomic and voluntary behaviors.

At present, it is unclear to what extent different rhythmic behaviors can be controlled by the same cerebellar region and cells, and if so, how they might contribute to synergistic control of the different motor domains involved. Here, we studied the activity of Purkinje cells in the lateral cerebellum in relation to respiratory control, while interfering with their whisker system. We focused on the lobules simplex, crus 1 and crus 2, because they are strongly related to rhythmic whisker movements and because their cells have been shown to respond to a variety of sensory inputs from the face, possibly integrating different sensorimotor behaviors (Bosman et al., 2010; Brown and Raman, 2018; Chen et al., 2016; Ju et al., 2019; Romano et al., 2018; Shambes et al., 1978). We found that Purkinje cells in these lobules co-modulate their firing rate with multiple phases of the respiratory cycle during unperturbed (eupneic) breathing. When we briefly stimulated the whiskers, the mice advanced the phase of their simple spike activity and breathing behavior concomitantly. The Purkinje cells that responded to whisker stimulation and also contributed to acceleration of respiration were particularly prominent in medial crus 1. Moreover, the respiratory adjustment following whisker stimulation could be induced by transiently stimulating these Purkinje cells in the lateral cerebellum optogenetically, whereas it was significantly reduced following Purkinje cell-specific impairment of postsynaptic AMPA receptors. Together, our data implicate that the cerebellar hemispheres can control respiratory behavior and align its rhythm with that of other behaviors in a phase-dependent manner, highlighting their putative role in synergistic integration of different sensorimotor activities.

## Results

### Unperturbed Respiratory Behavior

To find out to what extent Purkinje cells in the cerebellar hemispheres encode eupneic respiration, which is a cycle of inspiration, post-inspiration, and expiration (Anderson and Ramirez, 2017; Richter and Smith, 2014), we studied Purkinje cell activity patterns in awake head-restrained mice. During inspiration, contractions of the diaphragm and external intercostal muscles generate a volume expansion of the lungs, while during post-inspiration, the inspiration muscles relax and laryngeal constriction muscles retard lung compression (Dutschmann and Paton, 2002). During active expiration, abdominal and internal intercostal muscles contract depending upon metabolic demand (Aliverti et al., 1997; Bianchi and Gestreau, 2009). The respiratory phases of awake head-restrained mice were measured with a pressure sensor placed under the abdomen and analyzed upon phase transformation. Under these conditions, the mice had a median breathing frequency of 2.4 Hz (interquartile range [IQR]: 1.0 Hz; n = 13 mice), with a median coefficient of variation (CV) of 0.51 (IQR: 0.35), indicating the fast nature and substantial level of variability of their breathing rhythm at rest (Figures S1A–S1C).

### Purkinje Cell Complex Spike Activity Peaks after Inspiration

**Figure 1:**
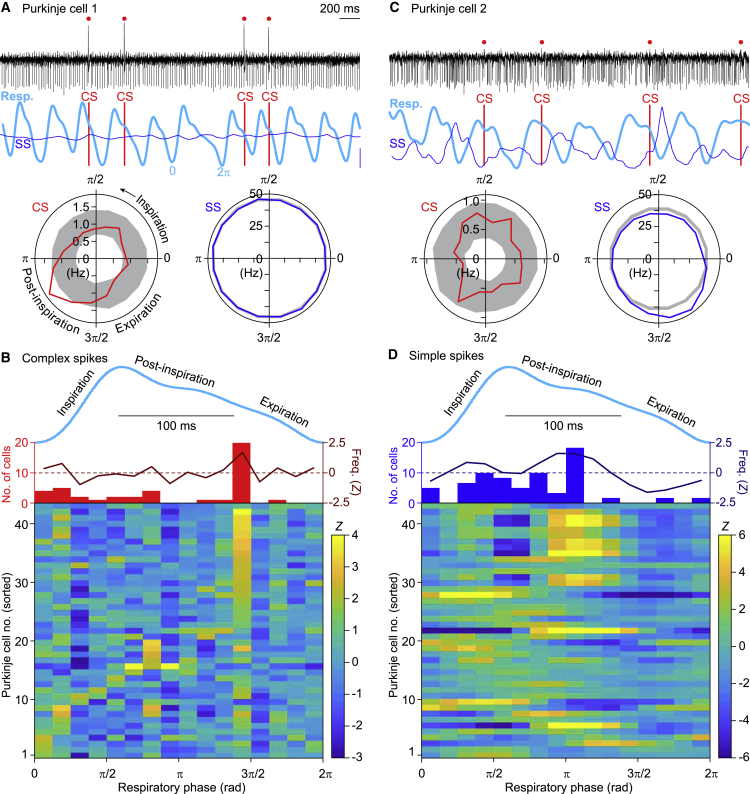
Purkinje Cells (PCs) in the Lateral Cerebellum Encode Eupneic Breathing For a Figure360 author presentation of this figure, see https://doi.org/10.1016/j.celrep.2020.107867. (A) An example recording of a PC showing complex spike (CS), but no simple spike (SS), modulation during unperturbed (eupneic) respiration in an awake mouse. The complex spikes are indicated by red dots and vertical lines. The instantaneous simple spike rate (thin blue line) is indicated in combination with the respiratory signal (thick cyan line). The scale bar on the right indicates 25 Hz of simple spike modulation. The raw signal indicates that the complex spikes preferably occurred around the transition from post-inspiration to expiration as confirmed by a polar plot summarizing the whole recording. The radial axes represent the spike frequency. The gray areas indicate the 99% confidence interval after bootstrap. (B) Of the 43 PCs recorded during eupneic respiration, 19 displayed their maximal complex spike firing around 3π/2. This is illustrated as the average modulation in firing rate (red line, middle), the distribution of the phases of strongest modulations (histogram; middle), and a heatmap illustrating the complex spike firing patterns of 43 PCs (bottom). This analysis was performed without pre-selection of PCs. For comparison, a randomly chosen respiratory cycle is indicated (cyan). Note that the respiratory trace is plotted based on time, while the heatmap and histogram are based on the phase. (C) An example of another PC, showing relatively weak complex spike modulation, but strong simple spike modulation during eupneic respiration. (D) The same analysis as in (B), but for the simple spikes, revealing a preference for simple spike firing during post-inspiration (just before the complex spike peak) and a relatively low firing rate during expiration (following the complex spike peak). Note that the PCs of both heatmaps are sorted by the phase of the maximal increase in complex spike firing. Consequently, the cell numbers of (B) and (D) refer to the same PCs. See also Figure S1.

In the first set of experiments, we quantified the complex spike firing rate modulation along the respiratory cycle at rest in awake mice by comparing the measured distributions of complex spikes along the breathing cycle with randomly shuffled complex spike distributions. For analysis of the experimental data, the entire epoch of recording was used without selecting only cycles including a complex spike, and the random shuffling was performed 500 times, upon which the 99% confidence interval (*Z* = 3) was calculated. Firing patterns exceeding this 99% confidence interval were considered indicative of a statistically significant modulation (Figure 1A). Of the 43 recorded Purkinje cells, 9 (21%) displayed significant modulation, but also many of the other Purkinje cells showed some degree of modulation (Figure S1E). In general, the maximal depth of the complex spike modulation did not correlate well with the average complex spike firing rate (r = 0.13, p = 0.396, n = 43 Purkinje cells, Spearman rank correlation; Figure S1F). Of all 43 Purkinje cells, 19 (44%) displayed maximum complex spike firing in the period just before 3π/2, which is around the transition from post-inspiration to expiration, whereas the other Purkinje cells typically peaked at a given phase during inspiration or post-inspiration, but not during expiration (Figure 1B; Figure S1G).

### Purkinje Cell Simple Spike Modulation Is Roughly Complementary to Complex Spike Activity

**Figure 2:**
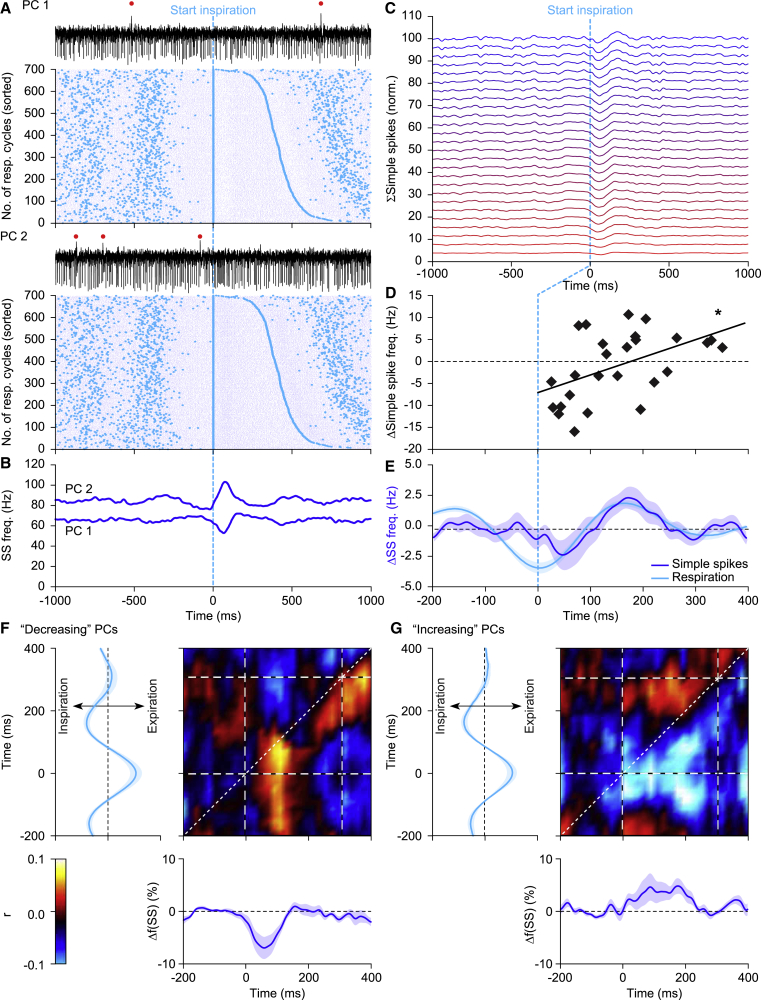
Eupneic Respiration Is Associated with Both Increased and Decreased Simple Spike Firing (A) During the respiratory cycle, simple spike (SS) modulation can either be predominantly decreasing or increasing, as illustrated by two representative PCs recorded simultaneously in the same animal. The raster plots are aligned on inspiration start and sorted according to the interval to the next inspiration. Large cyan dots indicate inspiration start and small blue dots the SSs. The red dots on top of the traces indicate complex spikes. (B) Convolved peri-stimulus time histograms of the two PCs shown in (A). (C) Stacked line plot of the instantaneous SS firing rates of all 26 PCs displaying a statistically significant SS modulation firing during unperturbed breathing. SS firing is displayed in percentage of baseline firing and normalized so that the upper line reflects the population average. The PCs are ordered from the strongest decrease (bottom, red line) to the strongest increase (top, blue line) in SS modulation. Each trace is aligned to inspiration start. (D) Scatterplot of moments of maximal modulation per PC, showing a correlation between the time of maximum modulation and its amplitude (r = 0.48, p = 0.012, Spearman rank correlation). Note that on average, as well as at individual cell level, the suppression of SSs preceded the increase. (E) Overall, the SSs were found to follow rather than to lead the respiration. (F) Correlation matrix showing a positive correlation between SS firing (blue trace [bottom] shows convolved peri-stimulus time histogram triggered on inspiration start) and respiration (cyan trace, left) based on trial-by-trial variance analysis in PCs that predominantly showed decreased SS firing rate linked to the respiratory cycle. Average of 13 PCs during unperturbed breathing. Note that the main simple spike activity follows the respiration (red area is below the 45° line). (G) Similar analysis of the 13 PCs that predominantly showed increased SS firing during the respiratory cycle, with mostly negative correlation. Thus, for both types of PCs, the correlation was opposite to their mode of modulation, indicating that the shallower the respiration, the stronger the SS modulation. Lines indicate averages and shaded areas SEM. See also Figure S2.

The median simple spike rate of the 43 Purkinje cells was 64.5 (IQR: 34.9) Hz (Figure S1D). The simple spike activity of the majority of these cells (i.e., 35% or 81%) showed a statistically significant modulation across different phases of respiration (Figure 1C; Figure S1E). Compared with the modulation of complex spike firing, the preferred phases of the peaks of the simple spike modulation were more closely associated with the inspiration and early post-inspiration periods (Figure 1D; Figure S1H). When we considered the absolute timing, rather than the phase, of simple spike modulation of single Purkinje cells relative to the start of inspiration, we found that the simple spike rate modulation of 26 Purkinje cells exceeded a Z criterion of higher than 2 (p < 0.05). Simple spike modulation was mostly bi-directional. In half of the cells the amplitude of the increase in simple spike modulation was stronger than that of the decrease, whereas in the other half it was opposite (Figures 2A–2C; Figure S2A). The peaks of decreased firing generally preceded those of increased firing, yielding a population average of a short-latency decrease followed by an increase of simple spike activity (Figures 2C–2E). This order of events of simple spike decreases and increases was substantiated by a positive correlation between the amplitude of the strongest correlation and its time of occurrence (r = 0.49, p = 0.012, n = 26, Spearman rank correlation; Figure 2D). The preference for decreased or increased firing did not depend on the baseline simple spike frequency (r = −0.14, p = 0.498, Spearman rank correlation; Figure S2B). The population average of simple spike activity approximated the actual respiratory behavior rather well with zero phase lag, suggesting the relevance of a population-encoding mechanism (Figure 2E).

We further explored whether trial-by-trial variations in simple spike firing correlated with variations in the respiratory signal. We designed a matrix of correlation in which, for each respiratory cycle, the respiratory signal was compared with the instantaneous simple spike rate aligned to the start of each inspiration. This analysis revealed the temporal relationships between both signals whereby a correlation along the 45° line indicates a synchronous event. Purkinje cells displayed correlations between simple spikes and respiration, irrespective of their preference for increased or decreased simple spike firing. The strongest effects were found with the respiratory signal leading the simple spike firing (Figures 2F and 2G). Thus, while simple spikes generally co-modulated with the phase of the respiratory signal with approximately a zero lag (Figure 2E), the depth of their simple spike modulation reflected the depth of the respiration with a delay.

The complex spike and simple spike modulation of each Purkinje cell typically occurred during different phases of the respiratory cycle, although often not in exact anti-phase (Figure S2C). The occurrence of increased simple spike firing around π correlated well with increased complex spike firing around 3π/2 (r = 0.536, p < 0.001, Spearman rank correlation; Figure S2D). In turn, this latter peak in complex spike firing correlated with a subsequent decrease in simple spike firing during expiration (r = −0.431, p = 0.004, Spearman rank correlation; Figure S2E). Thus, there were signs of reciprocity between complex spike and simple spike firing with a temporal shift of about 50–80 ms, which is reminiscent of studies of other cerebellar regions (Badura et al., 2013; Chaumont et al., 2013; Witter et al., 2013).

### Whisker Stimulation Increases the Probability of a Phase Reset of Inspiration

**Figure 3:**
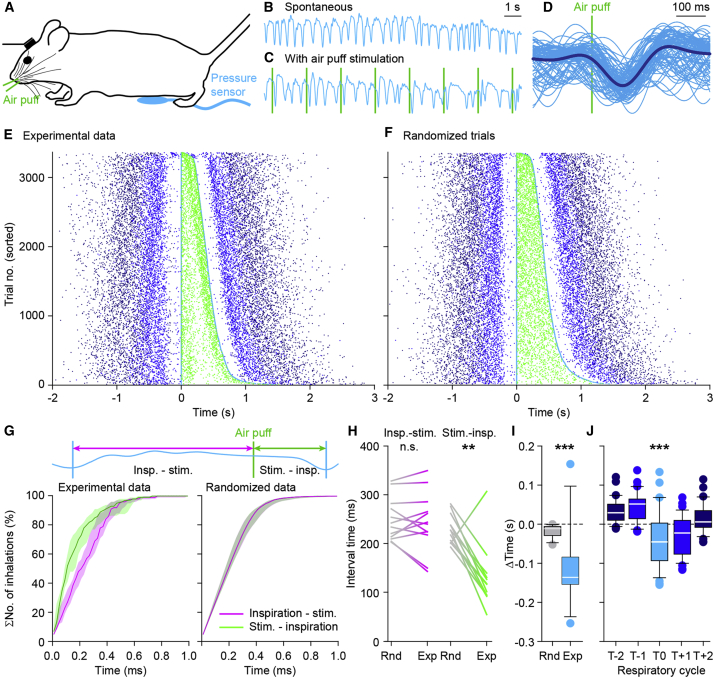
Whisker Pad Stimulation Triggers Inspiration (A) Every 2 s, mice received an air puff to their whisker pad while their respiration was recorded using an abdominal pressure sensor. (B and C) In comparison with the relatively regular breathing in the absence of air puff stimulation (B), the respiratory pattern appeared to be affected by sensory stimulation (C). Whisker pad stimulation (vertical lines) often triggered inspiration. (D) The raw respiratory signals around the air puff (90 trials of the same experiment as in C with the average [thick line] overlaid) demonstrate that whisker pad stimulation often triggers inspiration. (E) Raster plots showing respiratory cycles from 12 mice pooled together and sorted based upon the duration of the respiratory cycle during which the air puff (light green dots) was applied. The trials were aligned on the start of the last inspiration before the onset of the stimulus. Cyan dots indicate the start of the last inspiration before and the first inspiration after the air puff. The previous and subsequent respiratory cycles are indicated by increasingly darker shades of blue (see color code of J). In this plot, the air puffs are concentrated just after or just before the start of an inspiration. The latter reflect the triggering of the next inspiration by the air puff. This effect was not observed when the stimulation occurred just after the start of inspiration. (F) Upon random shuffling of the respiratory cycles, the air puffs are equally distributed over the respiratory cycle. (G) Cumulative distributions of 57 epochs (of approximately 100 trials each) originating from 12 mice demonstrate that the interval between the air puff and the start of the next inspiration was shortened relative to the interval between the start of the previous inspiration and the air puff (not visible in randomized data). Lines are medians, and shaded areas indicate the interquartile ranges. (H) The anticipation of the start of the next inspiration was also obvious from the median intervals per mouse and comparing the interval between the start of the preceding inspiration and the air puff (left) with the interval between the air puff and the start of the next inspiration (right) between randomized (R.) and experimental (E.) data. The former interval did not differ between the randomized and the experimental data (p = 0.937), whereas the latter did (^∗∗^p < 0.01, Wilcoxon signed rank tests). (I) Differences between the intervals between inspiration and puff versus those between puff and next inspiration (***p < 0.001, two-way ANOVA). (J) Boxplots of the duration of the respiratory cycles around the puff indicated that indeed the cycle during which the whisker pad stimulation was given was shorter. T0 is the cycle during which the air puff was given (***p < 0.001, repeated measures ANOVA). See also Figure S3.

Given the intricate relationships between orofacial behaviors in general and the harmonization of respiratory and whisking behavior in rodents in particular (Kurnikova et al., 2017; Lu et al., 2013; Moore et al., 2013), we wondered how an air puff to the facial whiskers that triggers reflexive whisker protraction (Bellavance et al., 2017; Brown and Raman, 2018; Romano et al., 2018) would also affect the respiratory cycle. To evaluate this, we subjected 12 mice to periodic 0.5-Hz whisker stimulation while measuring their respiration (Figures 3A–3D). When delivered within 100 ms after the start of the previous inspiration, the air puff had little effect, but otherwise it accelerated the start of the next inspiration with a median latency of 91 (IQR: 106) ms (Figure 3E; Figure S3A). Thus, stimulation of the whiskers induced not only a response in whisker movements, but also a phase-dependent accelerating respiratory response, shortening the interval between the air puff and the start of the next inspiration. We tested the specificity of the impact of puff stimulation on the timing of inspiration by comparing experimental with randomized data (p < 0.001, degrees of freedom [df] = 1, *F* = 15.993, interaction effect, two-way ANOVA after log transformation) and found this effect to be significant for the inspiration following the stimulus, but not for the preceding inspiration (p < 0.001, *t* = 5.220, df = 11 and p = 0.471, *t* = 0.746, df = 11, respectively, paired t tests, α_corr_ = 0.025 after Bonferroni correction; Figures 3G–3I). There was also some asymmetry in the randomized data (Figure 3I), reflecting the skewness of the distribution of respiratory intervals and the larger chance of giving a stimulus during a relatively long interval. Rather than entraining their respiratory rhythm to the frequency of air puff stimulation, the mice adjusted the timing of inspiration during the respiratory cycle directly following sensory stimulation (Figure 3J; Figures S3B and S3C). Variations in the level of sensory-induced whisker protraction and depth of respiration were correlated; trial-by-trial variations revealed that stronger whisker protractions preceded deeper levels of respiration, confirming the relationships between different orofacial behaviors in mice (Figure S3D).

### Purkinje Cells Sensitive for Whisker Stimulation Jointly Encode Sensory Stimulus and Motor Output

Given that Purkinje cells in the lateral cerebellum respond to whisker stimulation (Bosman et al., 2010; Brown and Raman, 2018; Romano et al., 2018) and modulate their firing rate along the respiratory cycle (Figure 1), we examined whether Purkinje cells could mediate the stimulation-induced change in respiratory timing. To this end, we compared the spiking pattern of 57 Purkinje cells during periodic whisker stimulation (Figures S4A–S4D).

**Figure 4:**
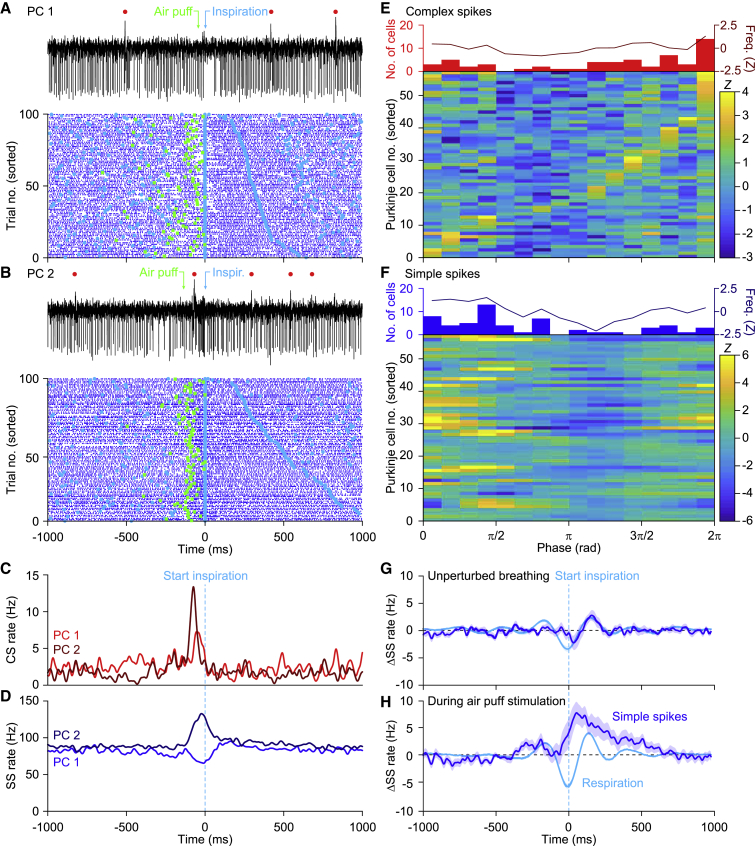
PC Activity Anticipates Respiratory Responses (A–F) Representative PC recordings showing either suppressed (A) or increased (B) simple spike firing upon whisker pad air puff stimulation. Above the trace, the complex spikes are indicated by red dots, the start of the air puff by a green arrow, and the start of inspiration by a cyan arrow. Below the traces are raster plots of simple spike activity aligned on the start of the first inspiration after the air puff. In the raster plots, the air puffs are indicated by green squares. The trials are sorted based on the duration of the respiratory interval following the air puff. The starting moments of inspiration are indicated by cyan dots. Note that the complex spikes are not shown in the raster plots for reasons of clarity. Convolved histograms of the complex spikes (C) and simple spikes (D) of these two illustrated PCs aligned on the first inspiration onsets after stimulus. The complex spike (E) and simple spike (F) data of the entire population of 57 PCs measured in this way are indicated in heatmaps. The PCs are sorted according to the moments of their maximal complex spike firing. (G and H) The same plot of Figure 2E is illustrated (G) for comparison along with the homologous plots for the air puff-induced anticipated inhalations (H). In both cases, a similarity can be observed between the profiles of the averaged respiratory signal and the averaged simple spike activity. In the latter case, the simple spikes modulation anticipated the averaged respiration signal. Lines indicate averages and shaded areas SEM. See also Figures S4 and S5.

First, we examined the firing pattern during the whole period with 0.5-Hz whisker pad stimulation. As during unperturbed respiration, complex spike and simple spike firing were modulated in tune with the respiratory cycle, but the timing of both complex spikes and simple spikes was now different (Figures 4A–4F). The temporal relation between simple spike and complex spike firing, as found during unperturbed respiration (Figures 1B and 1D; Figures S2C–S2E), was disrupted and no longer significant during the whole period with 0.5-Hz whisker stimulation (p > 0.05 for simple spike firing in all bins compared with the bin with the strongest complex spike modulation, Pearson correlation tests with Benjamini-Hochberg correction for multiple comparisons; Figures 4E and 4F).

**Figure 5:**
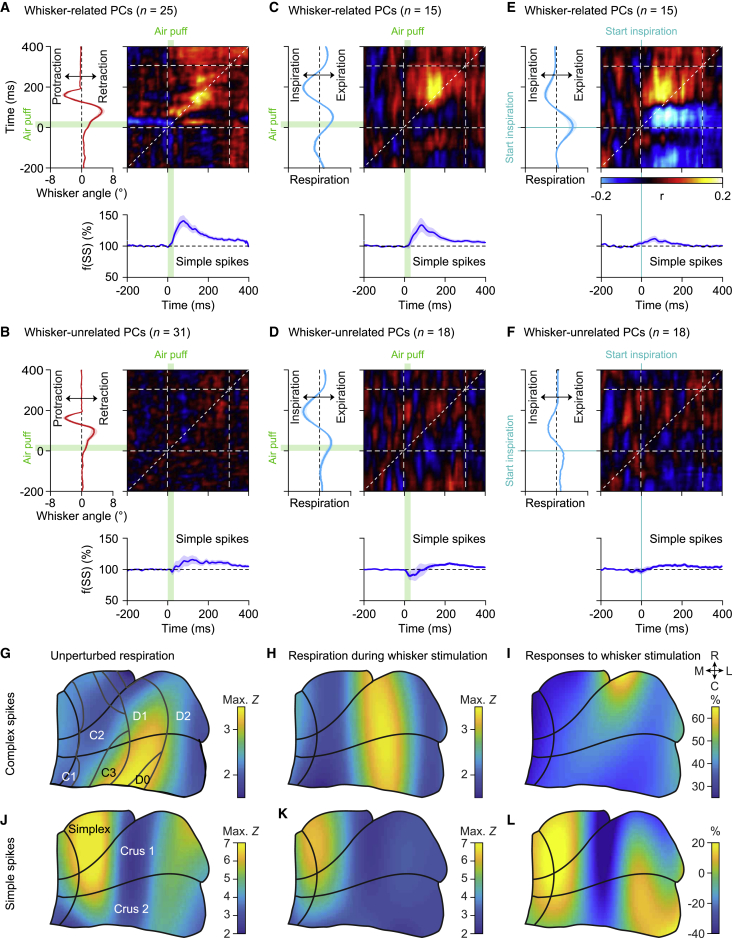
PC Encoding of Whisker Movements and Respiration Is Related in Time and Space (A–F) Correlation matrix between simple spike firing (blue trace at the bottom represents the averaged convolved peri-stimulus time histograms) and mean reflexive whisker protraction (red trace on the left) for PCs with (A) (n = 25) and without (B) (n = 31) significant correlation between simple spike firing and air puff-induced whisker movement (see STAR Methods). For 15 out of the 25 whisker-related cells and 18 out of 31 whisker-unrelated cells, the respiratory signal was simultaneously recorded and used for the respiration-spike matrix of correlation in (C) and (D), respectively. The whisker-related PCs had a higher correlation between their instantaneous simple spike rate and respiration than the other PCs (*U* = 67, p = 0.013, Mann-Whitney test). The location of the maximal correlation above the 45° line indicates that in trials in which the PCs fired more simple spikes, then a few tens of milliseconds later, the amplitude of the respiration was bigger and vice versa. In addition, the simple spike to whisker correlation (for the whisker-related cells) is stronger and earlier in time when the matrix of correlation is aligned to the puff-induced inhalation, rather than to the puff itself (E). Conversely, on average, the whisker-unrelated cells did not show a clear correlation even when the signals were aligned to the air puff-induced inspiration (F). Shaded areas around the traces indicate SEM. (G) Based upon the entry point of each electrode relative to a standardized map of the simplex, crus 1, and crus 2 lobules, a map was created indicating the spatial distribution of the maximal increase (in *Z* score) of complex spikes during the respiratory cycle in the absence of whisker pad stimulation. The tentative locations of the cerebellar modules are indicated. (H and I) The same during the presence of whisker pad stimulation (H). This analysis revealed an area with a relatively strong correlation between respiration and complex spike firing in the medio-lateral part of crus 2 extending rostrally in crus 1. These could be partially explained by the pattern of response probabilities (in percentage of baseline firing) to whisker pad air puff stimulation (I). (J–L) The same as (G), (H) and (I), respectively, but now for simple spikes. C, caudal; L, lateral; M, medial; R, rostral. See also Figure S4.

When we related simple spike modulation to the respiratory rhythm following whisker stimulation, we found that 20 out of the 32 (62%) Purkinje cells with a significant simple spike modulation predominantly increased their simple spike activity, whereas 12 (38%) predominantly decreased their simple spike firing. Compared with unperturbed respiration, the population increase of simple spike firing now peaked during earlier phases of the respiratory cycle, pointing toward an acceleration in their activity (*U* = 922.5, p = 0.035, Mann-Whitney test; Figures 4G and 4H). Restricting the analysis to the cycle around the air puff, it became apparent that the population average of simple spike firing now preceded the change in respiratory behavior, suggesting that air puff-triggered simple spike responses could contribute to the observed acceleration of inspiration. We further examined this possibility by performing a trial-by-trial analysis of variation. During eupneic breathing, the prevalence of correlation was below the 45° line for both suppressive and facilitating Purkinje cells, indicating that under these circumstances the simple spike modulation follows respiration and therefore cannot control it. However, during perturbed respiration, the modulation of simple spike firing preceded the ongoing respiration by a few tens of milliseconds (Figures 5A–5F). Moreover, when we segregated the cells that showed simple spike modulation to whisker stimulation (Figure 5C) from those that did not (Figure 5D), we observed that the maximal correlations between respiration and simple spike firing were stronger in the whisker-related than in the non-whisker-related Purkinje cells (median [IQR]: r = 0.30 [0.09] versus 0.23 [0.10]; *U* = 67, p = 0.013, Mann-Whitney test). These data confirmed that simple spike responses following whisker stimulation are endowed with the temporal features for accelerating respiratory responses, whereby the simple spike responses predict the strength of the inspiration.

To further substantiate the correlation between simple spikes, whisker movement, and respiration at single-cell level, we compared the maximal correlation between trial-by-trial variations in instantaneous simple spike rate and whisker movements with that between simple spike rate and respiration. We found that Purkinje cells whose fluctuations in the simple spike rate correlated well with whisker movement preferentially also showed a correlation between fluctuations in simple spike rate and inspiration (r = 0.44, p = 0.010, Spearman rank correlation; Figure S4E).

During perturbed respiration, about half of the Purkinje cells showed their peak in complex spike activity during the last quarter of the respiratory cycle, corresponding to expiration (Figures 4A–4C and 4E), with the strongest peak in complex spike firing occurring around 40 ms after whisker pad stimulation (Figures S5A and S5B), and thus approximately 50 ms before the average start of the first inspiration after the stimulus (Figure 4E). To test the possibility that the complex spikes can contribute to the acceleration of the next inspiration, we compared the timing of complex spike firing during individual trials relative to that of the start of the inspiration. However, we found no clear relation between them (Figure S5A). Accordingly, when we compared trials with and without complex spikes, we could not find any obvious difference in the timing of the next or subsequent start of inspiration (Figures S5C–S5E). Thus, we conclude that the complex spikes observed in the lateral cerebellum, although reacting to whisker stimulation, do not modulate the timing of respiratory responses to whisker pad stimulation in the short term.

### Respiration-Related Purkinje Cells Are Located in Specific Portions of the Cerebellar Cortex

Next, we mapped the location of the Purkinje cells recorded in this study. During unperturbed respiration, the strongest complex spike modulation was found laterally in crus 2 (Figure 5G). This complex spike hotspot extended rostrally into crus 1 during respiration perturbed by whisker stimulation (Figure 5H). The Purkinje cells in crus 1 that were recruited during perturbed, but not during unperturbed respiration, were mainly those that responded with a complex spike response directly to the sensory stimulation (Figure 5I). Of the 57 recorded Purkinje cells, 53 (93%) responded with a statistically significant complex spike response (Figure S4D). The simple spike responses showed a distribution that was largely complementary to that of the complex spike responses. The Purkinje cells with a predominantly increased simple spike rate during an unperturbed respiratory cycle were largely found around the border between the vermis and the simple lobule and crus 1, with a few cells extremely lateral in crus 1 (Figure 5J). The cells that showed decreased simple spike firing during unperturbed respiration were largely confined to a parasagittally oriented strip in the middle of crus 1 and crus 2. During perturbed respiration, this pattern was largely unaltered, although the lateral regions now also showed a decreased simple spike firing rate (Figure 5K). Importantly, the Purkinje cells in medial crus 1 showed particularly strong correlations to both whisker inputs and respiration (Figure 5L).

### Purkinje Cell Stimulation Mimics the Impact of Whisker Stimulation on Respiratory Timing

**Figure 6:**
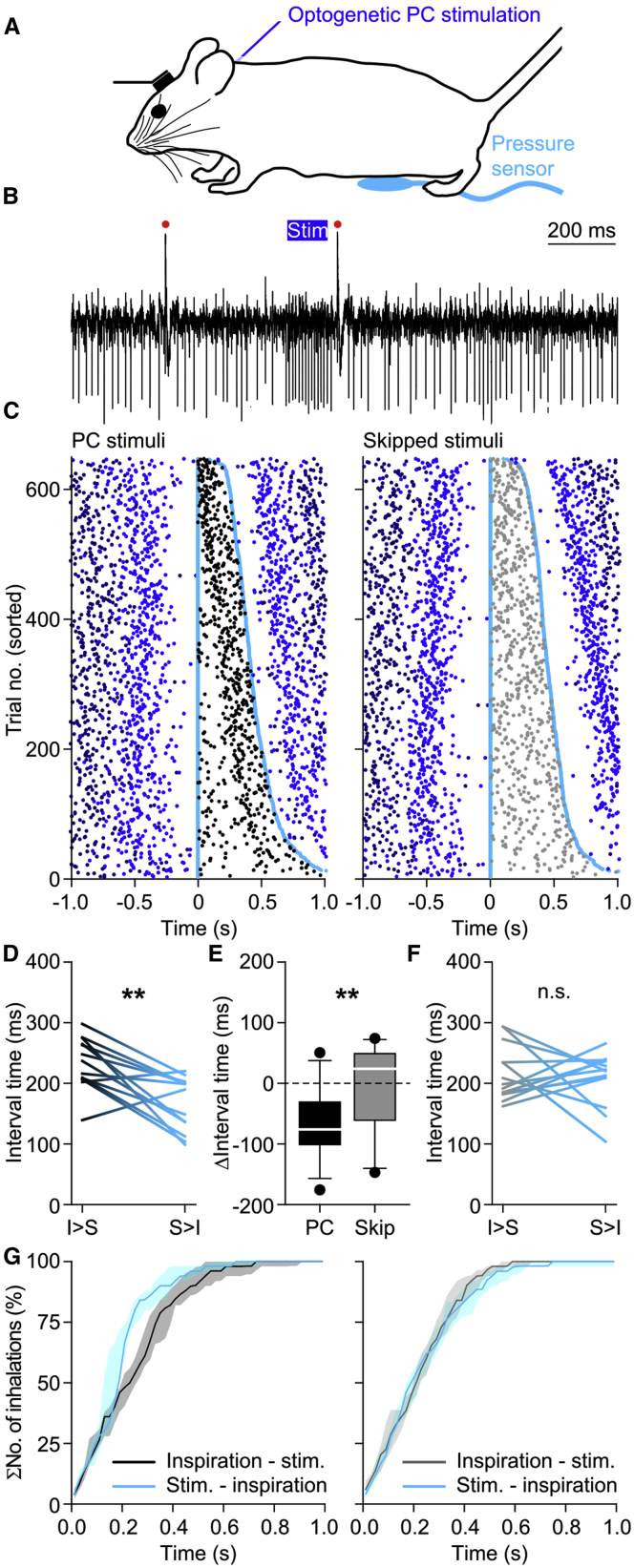
Optogenetic Stimulation of PCs Triggers Accelerated Inspiration (A) The impact of optogenetic stimulation on respiratory timing was studied using transgenic mice expressing ChR2 exclusively in their PCs. (B) 100-ms blue light pulses caused brief increases in simple spike firing. Red dots indicate complex spikes. (C) Raster plots showing respiratory cycles from 13 mice pooled together and sorted based upon the duration of the respiratory cycle during which optogenetic PC stimulus (black dots, left) or no stimulus (skipped stimuli, gray dots, right) was applied. The trials were aligned on the start of the last inspiration before the onset of the stimulus. Cyan dots indicate the start of the last inspiration before and the first inspiration after the stimulus. The previous and subsequent respiratory cycles are indicated by increasingly darker shades of blue. Inspiration typically started around 200 ms after the onset of PC stimulation. (D) Optogenetic PC stimulation resulted in anticipation of the next inspiration, as the median interval between the stimulus and the start of the next inspiration was shorter than that between the start of the previous inspiration and the stimulus (^∗∗^p < 0.01, paired t test). (E and F) This effect was not present during trials without optogenetic stimulation, resulting in a significant difference between trials with (E) and without (F) optogenetic stimuli (^∗∗^p < 0.01, interaction effect, two-way ANOVA). (G) Cumulative histograms of the intervals between the start of inspiration and the stimulus (gray) and between the start of the stimulus and the start of the next inspiration (cyan). PC stimulation (left), but not skipped stimulation (right), accelerates the start of the next inspiration. The cumulative histograms show the medians of the distributions per mouse (around 100 trials in 13 mice). The shaded areas indicate the interquartile ranges. See also Figures S6 and S7.

Because our analyses revealed that during perturbed respiration increased simple spike firing preceded the accelerated inspiration, we wondered whether we could mimic the impact of whisker pad air puff stimulation on the timing of inspiration by transiently stimulating the Purkinje cells in the medial parts of lobule simplex, as well as the crus 1 and crus 2 areas highlighted above. To this end, we made use of transgenic mice that expressed channelrhodopsin-2 (ChR2) specifically in their Purkinje cells (*Pcp2-Ai27* mice) (Romano et al., 2018; Witter et al., 2013). In line with previous whole-cell recordings *in vivo* (Witter et al., 2013), a brief pulse of blue light triggered a strong increase in simple spike firing (Figures 6A and 6B). We randomly intermingled trials during which optogenetic stimuli were either applied or skipped. The latter trials served to exclude synchronization between stimuli and respiration. Purkinje cell optogenetic stimulation significantly accelerated the occurrence of the next inspiration (p = 0.001, *t* = 4.286, df = 12, paired t test). This effect did not occur in the trials in which the stimuli were skipped (p = 0.650, *t* = 0.466, df = 12, paired t test, α_corr_ = 0.025 after Bonferroni correction): the inspiration started 189 ms (median, IQR: 243 ms) after the onset of optogenetic stimulation compared with 224 ms (IQR: 212 ms) following the skipped stimuli, which was significantly different (p = 0.006, df = 1, *F* = 8.423, interaction effect, two-way ANOVA on power transformed data; Figures 6C–6F). The same optogenetic stimulus triggered inspiration, as well as whisker protraction (Figure S6), supporting the idea that Purkinje cells in the same region of the cerebellar hemispheres can affect respiration and whisker movements.

To investigate whether the onset of the increased simple spike firing during optogenetic stimulation induced the accelerated inspiration rather than the rebound firing in the cerebellar nuclei that followed the offset of the optogenetic stimulation, we compared optogenetic stimulation of 100 ms with that of 200 ms in duration. Unlike whisker movements that are facilitated by rebound firing in the cerebellar nuclei (Brown and Raman, 2018; Proville et al., 2014), we did not observe a 100-ms delay when comparing 200- and 100-ms stimulation (Figure S7). To control for putative direct effects of light stimulation not involving optogenetic stimulation of Purkinje cells, we repeated the experiments in Cre-negative mice that did not express the optogenetic protein. As expected, we could not identify any sign of a respiratory response to light stimulation alone in these mice (*Z* = −0.734, p = 0.463, n = 5 mice, Wilcoxon signed rank test; Figures S6B and S7B), suggesting that the results described above are indeed mediated by Purkinje cells. We conclude that the onset of simple spike increases of the Purkinje cells in lobule simplex, as well as in crus 1 and crus 2 areas, is sufficient to induce an acceleration in the occurrence of the next respiratory cycle.

### Modification of AMPA Receptors at Parallel Fiber to Purkinje Cell Synapse Cancels the Impact of Whisker Stimulation on Respiratory Timing

**Figure 7:**
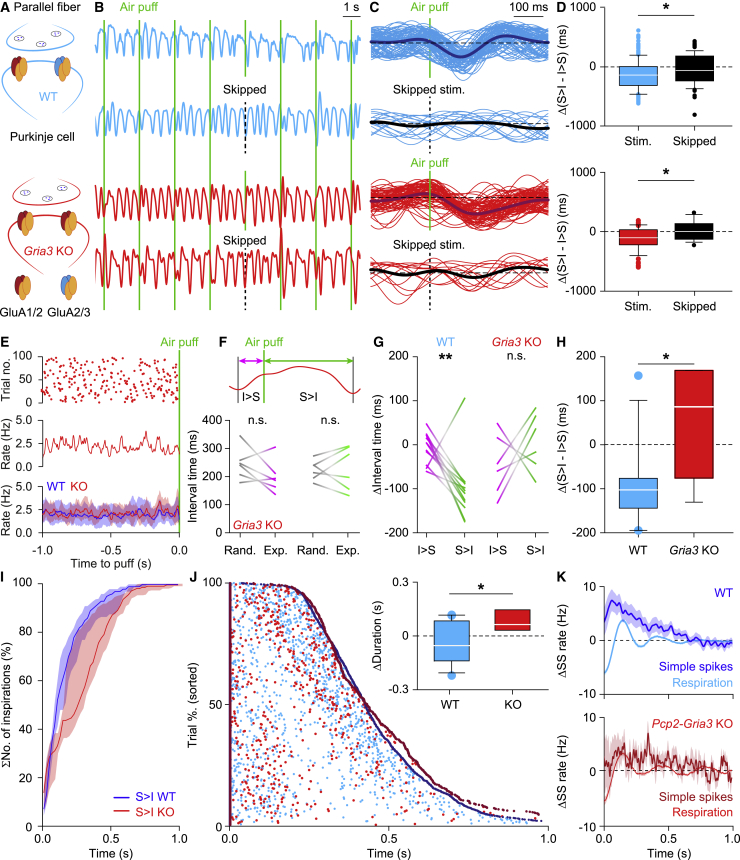
Impaired Respiratory Response in *Pcp2-Gria3* Knockout (KO) Mice (A) *Pcp2-Gria3*^−/−^ mice lack the gene for AMPA receptor GluA3 subunit specifically in PCs, which, as a result, express exclusively GluA1/GluA2-type AMPA receptors. (B) In *Pcp2-Gria3* KO mice, the whisker stimulation (vertical green lines) appeared to be less effective in triggering accelerated inspiration than in wild-type mice. To control for putative entrainment, we skipped around 15% of the stimuli in a subset of experiments. (C) The respiratory pattern of an exemplary *Pcp2-Gria3* KO mouse (red lines) around the air puff shows delayed inspiration when compared with a wild-type mouse (the cyan lines are the same as in Figures 3C and 3D). The respiratory patterns of the same mice around the skipped stimuli are shown in the lower graphs. (D) The air puff stimuli affected the differences between the intervals from inspiration (I) to stimulation (S) and those between the stimulation and the next inspiration in trials with, but not without, air puff stimulation; the data are normalized relative to randomized data (WT: p = 0.011, df = 1, *F* = 6.500, KO: p = 0.034, df = 1, *F* = 4.654, two-way ANOVA). (E) Raster plot and instantaneous respiratory frequency of an exemplary KO mouse, and the median instantaneous respiratory rate of all 6 KO mice tested and compared with 12 wild-type littermates (shades: interquartile range) during the 1 s before stimulation, showing no systematic entrainment to the periodic air puff stimulation. (F) The lack of entrainment was also reflected in the absence of a difference in the time intervals between the start of the last inspiration before the air puff stimulus in the experimental data in comparison with randomized data (p = 0.158, *t* = 1.660, df = 5, paired t test). Moreover, the intervals between the stimulus and the next inspiration were also not statistically significantly different between experimental data from the KO mice and randomized data (p = 0.819, *t* = 0.241, paired t tests, n = 6 mice). (G) The lack of an effect of air puff stimulation on the timing of the consequent inspiration in the *Pcp2-Gria3*^−/−^ mice (p = 0.285, df = 1, *F* = 1.205, interaction effect, two-way ANOVA) differed from that in their wild-type littermates. (H) The differences as plotted in (G) differed themselves between wild-type and *Pcp1-Gria3* KO mice (p *=* 0.011, *F* = 8.000, df = 1, interaction effect, two-way ANOVA). (I) Whereas wild-type mice accelerated the start of the next inspiration after whisker pad air puff stimulation (the blue line/area is a copy of the green line/area in Figure 3G, left panel), *Pcp2-Gria3* KO mice (red line/area) did not. Graphics represent cumulative distributions of the intervals between the stimulus and the start of the next inspiration, constructed of 100 trials per mouse (n = 6 mice). The lines show the medians and the shaded areas the interquartile ranges. (J) Raster plot showing the respiratory cycles perturbed by the air puffs sorted from the shortest (top) to the longest (bottom) duration. The beginning and the end of each cycle are represented with dark blue dots for the wild-type and dark red dots for the *Pcp2-Gria3* KO mice, while the relative time of the air puffs is depicted in cyan and red, respectively. Looking at all of the individual data points, both red and cyan dots are not randomly distributed and tend to accumulate before the subsequent inhalation. The delay to the start of the next inspiration is longer in *Pcp2-Gria3* KO than in wild-type mice, in line with the results plotted in (I). The impact of the puff on the overall duration of the cycle was different among wild-type and *Pcp2-Gria3* KO mice (p = 0.028, df = 1, *F* = 5.544, df = 1, interaction effect, two-way ANOVA; inset). (K) The simple spike activity of intact PCs of wild types increased during the air puff-triggered inspiration. This increase resembled and preceded the ongoing respiration. Conversely, the simple spike activity of the *Pcp2-GriA3* KO mice modulated differently than in wild-type mice and did not resemble the respiration signal. Lines indicate averages and shaded areas SEM.

To find out whether functionally intact cerebellar Purkinje cells are necessary for the respiratory changes induced with whisker stimulation, we investigated this response in a mouse model that lacked the AMPA GluA3 subunit at their parallel fiber to Purkinje cell synapses (Gutierrez-Castellanos et al., 2017) (Figure 7A). These *Pcp2-Gria3*^−/−^ mice have been shown to be impaired in simple spike modulation following whisker stimulation (Romano et al., 2018). *Pcp2-Gria3*^−/−^ mice showed a normal frequency of respiration during unperturbed respiration (median [IQR]: 2.6 [0.5] versus 2.4 [1.0] Hz for wild-type mice; p = 0.713, *t* = 0.378, df = 10, t test; Figure 7B). As their wild-type littermates (Figure S3), *Pcp2-Gria3*^−/−^ mice did not show consistent signs of entrainment (Figures 7C–7E). However, the mutant mice were impaired in their ability to advance the respiratory response following whisker stimulation. Whereas the experimental data from wild-type mice showed anticipated inspiration after the puff compared with randomized data (Figures 3H and 3I), those of *Pcp2-Gria3*^−/−^ mice did not (p = 0.285, df = 1, *F* = 1.205, interaction effect, two-way ANOVA; Figure 7F), revealing a significant difference in the impact of whisker stimulation between wild-type and *Pcp2-Gria3*^−/−^ mice (p = 0.011, df = 1, *F* = 8.000, interaction effect, two-way ANOVA; Figures 7G–7J). Moreover, the timing of simple spike rate modulation of the mutant Purkinje cells was significantly different from that in the wild-types in that they did not precede respiration directly following whisker stimulation (p = 0.040, *Pcp2-Gria3*^−/−^ versus wild-type, Wilcoxon rank-sum test; Figure 7K). These data indicate that a cerebellar, cell-specific interference with a mechanism that has the potential to increase the simple spike firing rate results in a hampered ability to accelerate the respiratory response.

## Discussion

Animals use periodic and oscillatory behaviors in a variety of functional movements, and they display adaptation in the coordination of such behaviors as a function of systematic changes of the environment. For instance, during exploration, rodents coordinate the movements of their whiskers with sniffing and breathing, and this coordination can be adjusted to the discrimination task involved (Kurnikova et al., 2017; Welker, 1964). The way the brain organizes such control mechanisms is largely unknown. Here, we show that during eupneic respiration, Purkinje cells in the simplex, crus 1, and crus 2 lobules fire in tune with specific phases of the respiratory rhythm. During unperturbed breathing, the activity of simple spikes and complex spikes can be described as efference copy of the respiratory signal, modulated by specific phases of the respiratory rhythm. In contrast, stimulation of the whiskers with air puffs accelerated simple spike activity that in turn contributed to a faster onset of inspiration. Our data show that increases in simple spike activity in the lateral cerebellum are probably sufficient to drive and integrate different forms of rhythmic behavior, because optogenetically driven increases in simple spike activity both accelerated the respiratory cycle in a phase-dependent manner and induced whisker protraction. Moreover, cell-specific blockage of GluA3-containing AMPA receptor signaling in Purkinje cells impaired the ability of the mice to advance both their simple spike response and their respiratory response following air puff stimulation of the whiskers, highlighting the necessity of an intact cerebellum for synergistic phase control. These responses are consistent with the idea that corollary discharges of multiple sources of respiratory signals are taken as predictions of the cycle’s phase, which, when disrupted, impute a recalibration of the respiratory cycle.

Isolated rhythmic behaviors are often controlled as muscle synergies by local networks. For instance, coordination between the left and right hindlimb can still take place during locomotion even after the descending connections from the motor cortex and brainstem to the spinal cord are disrupted (Desrochers et al., 2019). Likewise, when cerebellar function is impaired, basic muscle activities of breathing and related orofacial behaviors can still take place (Bellavance et al., 2017; Chen et al., 2005; Gewaltig and Diesmann, 2007). Thus, local networks in spinal cord and brainstem are sufficient to generate basic antagonistic muscle activities that mediate rhythmic properties of locomotion and breathing, respectively (Bellavance et al., 2017; Kurnikova et al., 2017; Talpalar et al., 2013; Tresch et al., 1999). However, the integration of multiple rhythmic behaviors requires cerebellar coordination, exemplified by paradigms requiring front and hindlimb movements, particularly in less stereotyped forms of locomotion and adaptation (Darmohray et al., 2019; Hoogland et al., 2015; Machado et al., 2015; Vinueza Veloz et al., 2015). Similarly, an intact cerebellum is required for adjusting the respiratory cycle during more complex tasks such as speech. We now provide evidence that changes in simple spike activity of Purkinje cells in the simplex, crus 1, and crus 2 areas, in which respiratory and whisking processing converge, contribute to re-adaptation of the respiratory timing signal following sensory perturbation of the facial whiskers. Natural or artificial activation of Purkinje cells sensitive for whisker stimulation can accelerate the occurrence of the next inspiration. The correlation between simple spike firing and respiration is particularly strong in the Purkinje cells whose simple spike firing co-modulate with whisker movement. In line with their instructive role following facial stimulation (Romano et al., 2018), optogenetic stimulation of Purkinje cells affects both respiration and whisker movements, providing synergistic control of two different forms of orofacial behavior.

Our study indicates that simple spike activity of individual Purkinje cells can simultaneously drive different forms of motor behavior, in this case, breathing and whisking. This finding elaborates on several behavioral studies demonstrating the role of the cerebellum in synergistic control of diverse motor domains (Albergaria et al., 2018). For example, the olivocerebellar system has been shown to be involved in the coordination between eye and hand movements (Kitazawa et al., 1998; Owens et al., 2018; Vinueza Veloz et al., 2015), between trunk and limb movements (Bakker et al., 2006; Caliandro et al., 2017), as well as between shoulder, arm, and finger movements (Thach et al., 1993; Timmann et al., 2000). The current study amasses to that lot by demonstrating the functional convergence of autonomic and sensorimotor behaviors on single Purkinje cells. Given their rich and diverse parallel fiber inputs mediating signals from different sensorimotor systems (De Zeeuw et al., 2011; Gao et al., 2012; Ito, 2000), we postulate that Purkinje cells in the cerebellar cortex mediate synergy and integration of different motor domains in both voluntary and autonomic systems.

The contribution of complex spikes to changes in respiratory behavior remains to be elucidated. They have a relatively low frequency, considerably lower than that of the respiratory cycle, but they tend to prefer specific phases across many cells, both with and without perturbation. Given the strong convergence of Purkinje cells upon cerebellar nuclei neurons, it is likely that multiple Purkinje cells thus encode specific moments of the respiratory cycle (see also Ju et al., 2019; Negrello et al., 2019). Nevertheless, comparing the acute respiratory responses during trials with and without complex spikes did not reveal any significant difference in behavior. We also could not find any differences in air puff-related inspiration between trials with and without complex spikes. This means that the phase-related signal conveyed by the complex spike is robust to the sensory perturbation. The only prominent difference was that the interval between peak activity of the complex spikes to that of the simple spikes robustly changed specifically in the perturbed trials, supporting a putative role in long-term plasticity (Coesmans et al., 2004; De Zeeuw and Ten Brinke, 2015; Gao et al., 2012; Suvrathan et al., 2016; Wang et al., 2000). Indeed, this possibility agrees with the fact that the complex spike frequency negatively correlates with the induction of long-term potentiation (LTP) at the parallel fiber to Purkinje cell synapse (Coesmans et al., 2004). It is also consistent with the observation that ablating this form of plasticity in *Pcp2-Gria3*^−/−^ mice corrupted the synergistic behavioral response following whisker stimulation. It will be interesting to investigate to what extent an induced shift in complex spike phase may have an impact on the relationship between different rhythms, including that of respiration.

It is likely that Purkinje cells in the cerebellar cortex influence the nuclei in the brainstem that control breathing and/or whisking. The cerebellar fastigial nucleus is known to modulate the respiratory cycle by sensing blood CO_2_ levels (Martino et al., 2006, 2007; Xu and Frazier, 2000; Xu et al., 2001). The roles of other cerebellar nuclei, which do not seem to sense CO_2_ levels (Xu et al., 2001), are controversial (Xu and Frazier, 2000). Possibly, the interposed nuclei play a role in the control of the upper airways, because bilateral lesions of this region suppress coughing responses (Xu et al., 1997). The central pattern generator for respiration is located in the pre-Bötzinger complex (Feldman et al., 2013; Moore et al., 2013; Ramirez et al., 2011; Smith et al., 1991). There is no direct connection from the cerebellar nuclei to the pre-Bötzinger complex nor to the adjacent Bötzinger complex (Teune et al., 2000), which is consistent with our finding that the Purkinje cell-mediated impact of whisker stimulation changes the timing, not the frequency, of respiration (Figure 3I). Possibly, cerebellar nuclei project, downstream of the pre-Bötzinger complex, to the region of the post-inspiratory complex at the border of the intermediate and gigantocellular reticular formation (Lu et al., 2013; Teune et al., 2000) and/or to the parabrachial complex, which projects to motor neurons of the diaphragm in the spinal cord (Dobbins and Feldman, 1994).

Thus, although the anatomical pathways via which the cerebellum could affect respiration are still a matter of debate, individual Purkinje cells in the lateral cerebellum can synergistically coordinate multiple motor behaviors, such as respiration and whisking, by injecting accelerating signals into diverging downstream circuitries.

## STAR★Methods

### Key Resources Table

**Table:** 

REAGENT or RESOURCE	SOURCE	IDENTIFIER
**Chemicals, Peptides, and Recombinant Proteins**		

Optibond adhesive	Kerr Corporation	33381E
Isoflurane	Pharmachemie	45.112.110
Rimadyl	Pfizer	CAS 53716-49-7
Lidocaine	Braun	RVG 07831
Buprenorphine	Reckitt Benckiser Pharmaceuticals	RVG 08725
Bupivacaine	Actavis	RVG 20949

**Experimental Models: Organisms/Strains**		

C57BL/6J mice	Charles River	IMSR_JAX:000664
*Tg(Pcp2-cre)2Mpin;Gt(ROSA)26Sor*^*tm27.1(CAG-OP4∗H134R/tdTomato)Hze*^	(Witter et al., 2013)	N/A
*Tg(Pcp2-cre)2Mpin;Gria3*^*tm2Rsp*^	(Gutierrez-Castellanos et al., 2017)	N/A

**Software and Algorithms**		

MATLAB	MathWorks	N/A
SpikeTrain	Neurasmus	N/A
BIOTACT Whisker Tracking Tool	http://bwtt.sourceforge.net	N/A
Custom whisker tracking code (accelerated version)	https://gitlab.com/neurocomputing-lab/whisker/bwtt-acceleration	N/A
Custom whisker tracking code (post-processing)	https://github.com/elifesciences-publications/BWTT_PP	N/A
Custom phase transform code	https://gitlab.com/neurocomputing-lab/whisker/extrac_inh_start	N/A

### Resource Availability

#### Lead Contact

Further information and requests for resources and reagents should be directed to and will be fulfilled with the Lead Contact, Laurens Bosman (l.bosman@erasmusmc.nl).

#### Materials Availability

This study did not generate new unique reagents.

#### Data and Code Availability

The custom code for the phase transformed can be found at https://gitlab.com/neurocomputing-lab/whisker/extrac_inh_start. The code for accelerated whisker tracking is available at https://gitlab.com/neurocomputing-lab/whisker/bwtt-acceleration. The previously published custom code complementing BWTT whisker tracking can be obtained via https://github.com/elifesciences-publications/BWTT_PP (Romano et al., 2018). Data are available upon reasonable request from the Lead Contact.

### Experimental Model and Subject Details

We used 18 WT adult mice with a C57BL/6J background (13 males and 5 females from Charles Rivers, Leiden, the Netherlands) for the electrophysiological recordings and compared their behavior to 13 *Tg(Pcp2-cre)2Mpin;Gt(ROSA)26Sor*^*tm27.1(CAG-OP4∗H134R/tdTomato)Hze*^ mice (Witter et al., 2013) expressing channelrhodopsin-2 (ChR2) for optogenetic stimulation of their Purkinje cells (6 males and 7 females from the same breeding colony as the WT mice, preferably using littermates). As controls for the optogenetic stimulation, we used five additional male Cre-negative *Gt(ROSA)26Sor*^*tm27.1(CAG-OP4∗H134R/tdTomato)Hz*^ mice. In addition, we used 6 *Tg(Pcp2-cre)2Mpin;Gria3*^*tm2Rsp*^ KO mice (3 males and 3 females) (Gutierrez-Castellanos et al., 2017) mice, also on a C57BL/6J background. The mice had an age of 4-7 months. Mice were socially housed until surgery and single-housed afterward with *ad libitum* access to food and water. The mice were kept at a 12/12 h light/dark cycle and had not been used for any other study before the start of the experiments described here. All mice were healthy and specific pathogen free (SPF). All experimental procedures were approved *a priori* by an independent animal ethical committee (DEC-Consult, Soest, the Netherlands) as required by Dutch law and conform the relevant institutional regulations of the Erasmus MC and Dutch legislation on animal experimentation. Permission was filed under the license numbers EMC3001, AVD101002015273 and AVD1010020197846.

### Method Details

#### Surgeries

All mice received a magnetic pedestal that was attached to the skull above bregma using Optibond adhesive (Kerr Corporation, Orange, CA) and a craniotomy that was made on top of crus 1 and crus 2. The surgical procedures were performed under isoflurane anesthesia (Pharmachemie, Haarlem, the Netherlands; 2%–4% V/V in O_2_). Post-surgical pain was treated with 5 mg/kg carprofen (“Rimadyl,” Pfizer, New York, NY), 1 μg lidocaine (AstraZeneca, Zoetermeer, the Netherlands), 50 μg/kg buprenorphine (“Temgesic,” Reckitt Benckiser Pharmaceuticals, Slough, United Kingdom) and 1 μg bupivacaine (Actavis, Parsippany-Troy Hills, NJ, USA). After three days of recovery, mice were habituated to the recording setup during at least 2 daily sessions of approximately 45 min. In the recording setup they were head-fixed using the magnetic pedestal. Further body movements were prevented by using a customized restrainer and filling the empty space with paper tissues.

#### Whisker pad stimulation and behavioral recordings

Sensory stimulation (0.5 Hz) was given to the center of the whisker pad of awake mice by means of air puffs given from approximately 5 mm at an angle of 30 degrees with the whisker pad. Each puff was around 2 bar and had a duration of 30 ms. Videos of the movements of the untrimmed large facial whiskers were made from above using a bright LED panel as back-light (λ = 640 nm) at a frame rate of 1,000 Hz (480 × 500 pixels using an A504k camera from Basler Vision Technologies, Ahrensburg, Germany). Respiration was recorded using a PowerLab 4/30 analog-to-digital converter (AD Instruments, Oxford, United Kingdom) in combination with a pressure sensor that was placed at the abdomen of the mice.

#### Electrophysiology

Electrophysiological recordings were performed in awake mice using quartz-coated platinum/tungsten electrodes (2-5 MΩ, outer diameter = 80 μm, Thomas Recording, Giessen, Germany). The latter electrodes were placed in an 8x4 matrix (Thomas Recording), with an inter-electrode distance of 305 μm. Prior to the recordings, the mice were lightly anesthetized with isoflurane to remove the dura, bring them in the setup and adjust all manipulators. Recordings started at least 60 min after termination of anesthesia and were made in lobules simplex, crus 1 and crus 2 ipsilateral to the side of the whisker pad stimulation at a minimal depth of 500 μm. The electrophysiological signal was digitized at 25 kHz, using a 1-6,000 Hz band-pass filter, 22x pre-amplified and stored using a RZ2 multi-channel workstation (Tucker-Davis Technologies, Alachua, FL).

#### Optogenetic stimulation

LED photostimulation (λ = 470 nm) driven by a Thorlabs LED driver (225 μW) was given through an optic fiber (400 μm in diameter, Thorlabs, Newton, NJ, USA). The optic fiber rested on the dura mater above the midline between crus 1, crus 2, approximately 1 mm lateral from the vermis, via the craniotomy. During experiments with optogenetic stimulation, trials without stimulation, with 100 ms and with 200 ms optogenetic stimulation were randomly intermingled.

#### Experimental design

During the experiments and formal analysis, the experimenters were blind to the genotype of the mice. All obtained data were included, provided the signal-to-noise ratio of the recordings allowed unbiased analysis. Regarding electrophysiological recordings, as an extra inclusion criterion we accepted only those recordings during which the amplitude and the width of the spikes were constant over time for correlation with the respiratory signal. The recordings in which the amplitude or the width of more three consecutive simple spikes exceeded three standard deviations above or below their average were considered unstable and excluded. In this way, any change in spike rate due to the instability of the recordings was avoided. Only single-unit recordings of Purkinje cells with a minimum recording duration of 120 s were selected for further analysis.

### Quantification and Statistical Analysis

#### Phase transformation of respiratory recordings

The signal from the abdominal pressure sensor was filtered with MATLAB’s (MathWorks, Natick, MA, USA) Butterworth bandpass filter (cut-off frequencies 1 and 10 Hz, chosen to include respiratory frequencies visible on the Fourier transform of the raw signal). For the averages of the respiration signal around the stimulus, movement artifacts were removed by excluding trials in which the signal surpassed three times the standard deviation in a 200 ms window before the stimulus. The phase transform of the respiration signal was acquired with the co_hilbproto (which calculates a ‘protophase’ of a scalar time series using the Hilbert transform) and co_fbtrT (protophase to phase transformation) functions from MATLAB toolbox DAMOCO. As the default setting, the DAMOCO toolbox chooses as initial phase the maximum of the respiration signal, but for our analysis it was more beneficial to set zero phase at the moment when the mouse starts inspiration. Therefore, before the phase transform the respiration signal was multiplied by −1, so that no changes needed to be made to the functions of this toolbox. Code is available at https://gitlab.com/neurocomputing-lab/whisker/extrac_inh_start.

#### Whisker movement tracking

The whisker movements were tracked as described previously (Ma et al., 2017; Rahmati et al., 2014; Romano et al., 2018) using the BIOTACT Whisker Tracking Tool (Perkon et al., 2011) in combination with an acceleration version of the BIOTACT code (https://gitlab.com/neurocomputing-lab/whisker/bwtt-acceleration and https://github.com/elifesciences-publications/BWTT_PP). Multiple acceleration strategies have been explored, resulting in speedups ranging from 2 × to 4,500 ×. Such speed improvements had a pivotal role in permitting us to analyze a large number 645 of whisker videos. For the purposes of this work, the whisker movements were captured as the average angle of all trackable whiskers per frame.

#### Electrophysiological analysis

Spikes were detected offline using SpikeTrain (Neurasmus, Rotterdam, the Netherlands). A recording was considered to originate from a single Purkinje cell when it contained both complex spikes (identified by the presence of stereotypic spikelets) and simple spikes, when the minimal inter-spike interval of simple spikes was 3 ms and when each complex spike was followed by a pause in simple spike firing of at least 8 ms.

#### Polar plots

Polar plots were generated to describe the correlation between respiratory phase and Purkinje cell spiking activity. To this end, we attributed each spike to a phase (using 16 bins) of the respiration and we compared the recorded distribution with a bootstrap analysis based upon a re-sampling of the spike times after shuffling the inter-spike intervals. The bootstrap analysis was repeated 500 times after which the 99% confidence interval was established. The *Z* score of each bin was derived by dividing, for each bin, the dividing the difference between number of spikes of a Purkinje cell during that bin and the average number of spikes of all bins by the standard deviation of all bins. This analysis focuses on the relative timing of spikes. Respiratory cycles during which no complex spike was fired are not represented in the polar plots.

#### Trial-by-trial correlation analysis

The inter-trial variations between the respiratory signal and the instantaneous simple spike firing rate (Figures 2F, 2G, and 5) or the average whisker angle (Figure 5; Figure S3D) were calculated and represented according to a previously published method (Romano et al., 2018; ten Brinke et al., 2015). Briefly, during each trial, the filtered respiration signal (see above) was compared to either the instantaneous simple spike rate or the relative whisker position without alignment to the baseline. The instantaneous simple spike rate was obtained by convolving spike occurrences across 1 ms bins with an 8 ms Gaussian kernel. The inter-trial variations were subsequently described by creating a matrix of Pearson correlation values for each 10 × 10 ms bin and visualized as heatmaps. In Figure 5, we separated between those Purkinje cells that had a significant correlation between their instantaneous simple spike rate and the whisker angle and those that had not. Significance was established by testing whether the correlation along the 45° line exceed the 99% confidence interval of a bootstrapped dataset in which the inter-spike times were randomly shuffled 500 times.

#### Sorted raster plots

To visualize the relation between stimulation and respiration (Figures 3E, 6C, 7J, and S3C) or between complex spike firing and respiration (Figure S5A), sorted raster plots were constructed. For each plot, a dataset composed of a balanced number of trials of all mice was generated and sorted based upon the duration of the respiratory cycle during which the stimulus was applied (Figures 3E, 6C, and 7J) or that of the respiratory cycle preceding the stimulus presentation (Figure S3C). The trials presented in Figure S5A were sorted based upon the interval from the air puff stimulus to the first complex spike following that moment. In Figure S5A, some of the Purkinje cells were recorded simultaneously, leading to a larger number of trials than in the other plots that are based upon mice. The experimental data were compared to a random shuffling of the durations of the respiratory cycles within each experiment (Figure 3F). During optogenetic stimulation, trials with and without stimulation were randomly intermingled and the trials during which the stimuli were skipped served as controls (Figure 6C). The differences between experimental and control data were substantiated by comparing the distribution of the intervals between the start of the last inspiration prior to stimulation and the moment of stimulation with the distribution of intervals between the stimulation and the start of the subsequent inspiration.

#### Anatomical maps

To visualize the distribution of the spike-respiration correlation respiration throughout the lobules simplex, crus 1 and crus 2 we developed an anatomical map of the distribution of the *Z* scored values obtained by the polar plots (Figures 5G, 5H, 5J, and 5K). Since the electrophysiological recordings were performed using a grid of 8 × 4 electrodes (placed always on the same type of craniotomy), we could retrieve the approximate location of each cell and plot the corresponding maximum *Z* score on an 8 × 4 matrix. Linear interpolation was used to smooth the edges of adjacent patches and the MATLAB function “imagesc” was eventually used to obtain the heating map that was overlapped to a schematic draw of the craniotomy. Similarly, also the air puff responses could be represented by plotting the values of maximum variation of firing rate of each cell (Figures 5I and 5L).

#### Statistics and visualization

Throughout the manuscript, data were tested for normality (one-sample Kolmogorov-Smirnov tests). If the data failed this test, we used either logarithmic or power transformation (depending on the shape of the distribution) before parametric testing or used non-parametric tests. Spike responses were considered statistically significant, if they exceeded a *Z* score of ± 3. Whenever applicable, two-sided tests were used. Unless stated otherwise, data are summarized as medians with the interquartile ranges. When applied, the method for the correction of multiple comparisons is indicated in the text, with α_corr_ the corrected threshold for significance.

Boxplots (e.g., see Figure 3J) indicate the distribution of the data with the box indicating the interquartile-range around the median (horizontal line). The whiskers indicate the 10^th^ and 90^th^ percentiles. Data points outside the 10^th^-90^th^ percentile range are indicated as separate dots. Violin plots (e.g., see Figure S1D) indicate the distribution of all data points as dots. The contours indicate a convolved histogram of the data points (along the y axis, using a Gaussian kernel and reflected along the vertical axis) and the horizontal lines show the 10^th^, 25^th^, 50^th^, 75^th^ and 90^th^ percentiles.

CV2 was calculated as follows:

$$CV2=2\left(\frac{\left|interval_{n+1}-interval_{n}\right|}{interval_{n+1}+interval_{n}}\right)$$

## Supplemental Information

Document S1. Figures S1–S7

Figure360. An Author Presentation of Figure 1

Document S2. Article plus Supplemental Information
