# Supplementary material for: Functional Convergence of Autonomic and Sensorimotor Processing in the Lateral Cerebellum
Source: Cell Rep. 2020 Jul 7;32(1):107867. doi: 10.1016/j.celrep.2020.107867 (PMC7351113; doi:10.1016/j.celrep.2020.107867)
Supplement: Document S1. Figures S1–S7 [file mmc1.pdf]

Cell Reports, Volume *32*

Supplemental Information

Functional Convergence of Autonomic and Sensorimotor Processing in the Lateral Cerebellum

Vincenzo Romano, Aoibhinn L. Reddington, Silvia Cazzanelli, Roberta Mazza, Yang Ma, Christos Strydis, Mario Negrello, Laurens W.J. Bosman, and Chris I. De Zeeuw

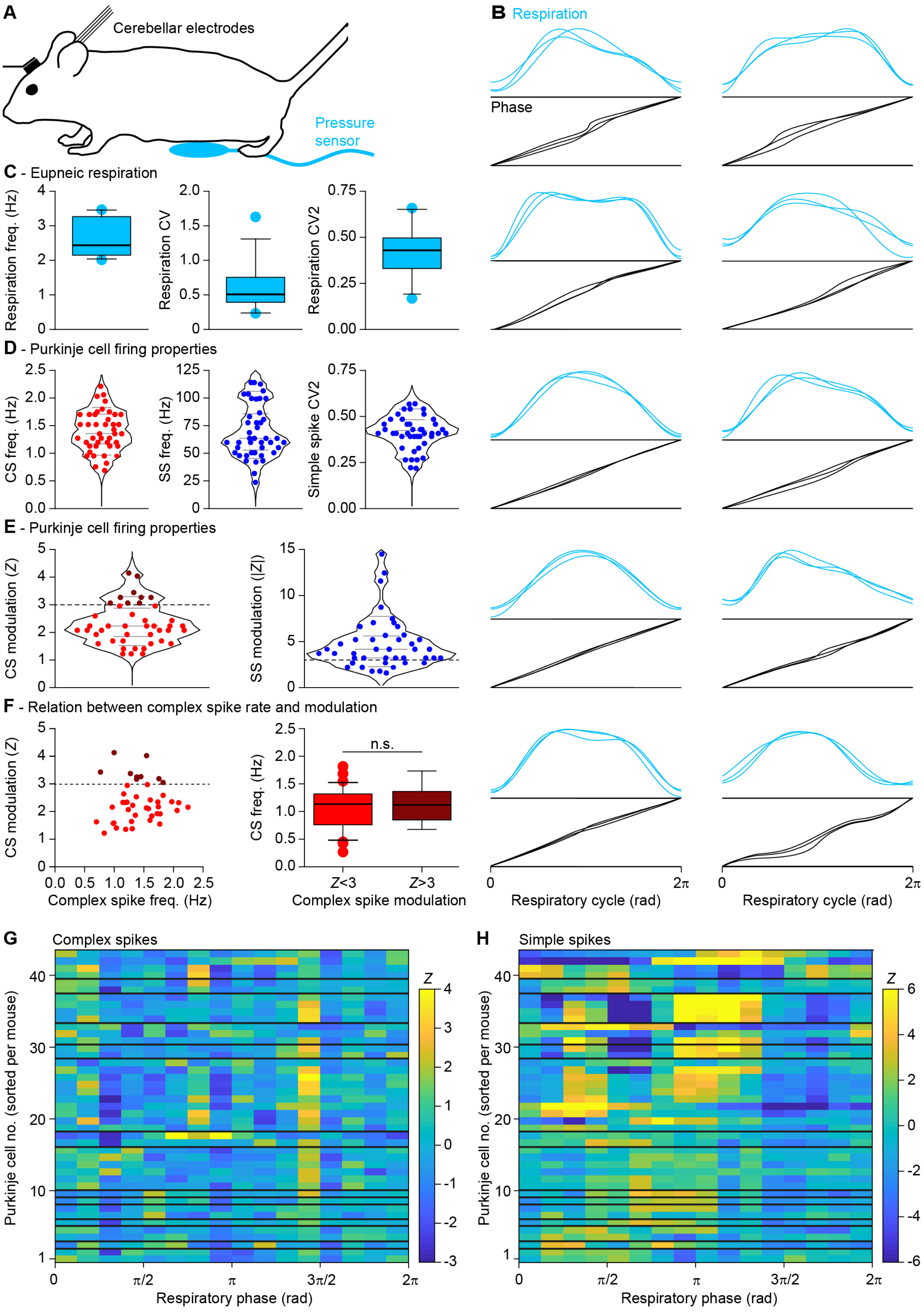

A
Cerebellar electrodes
Pressure sensor
B Respiration
Phase
C - Eupneic respiration
Respiration freq. (Hz)
Respiration CV
Respiration CV2
D - Purkinje cell firing properties
CS freq. (Hz)
SS freq. (Hz)
Simple spike CV2
E - Purkinje cell firing properties
CS modulation (Z)
SS modulation (|Z|)
F - Relation between complex spike rate and modulation
CS modulation (Z)
Complex spike freq. (Hz)
n.s.
CS freq. (Hz)
Z<3
Z>3
Complex spike modulation
Respiratory cycle (rad)
Respiratory cycle (rad)
G
Complex spikes
Purkinje cell no. (sorted per mouse)
Respiratory phase (rad)
Z
H
Simple spikes
Purkinje cell no. (sorted per mouse)
Respiratory phase (rad)
Z

Figure S1 – Purkinje cells in the lateral cerebellum encode eupneic breathing, Related to Fig. 1
A Single-unit recordings of Purkinje cells were made in the lobules simplex, crus 1 and 2 of awake, head-fixed mice during quiet, unperturbed (eupneic) respiration. **B** The pressure on the abdominal sensor was used as the raw respiratory signal (cyan). As the course and duration of each cycle could be quite variable, we used a phase transform (black) to obtain the instantaneous phase at each moment of the respiratory cycle. For ten mice, we show here three overlaid randomly selected cycles (during unperturbed breathing) with underneath it the three phase transforms. The three parts of the cycle, inspiration (starting at phase 0), post-inspiration and expiration can be seen in most traces. **C** Frequency, coefficient of variation (CV) and mean local coefficient of variation (CV2) of eupneic respiration in 13 mice. **D** The average complex spike (CS) and simple spike (SS) frequencies as well as the mean local coefficient of variation (CV2) of the simple spikes of 43 Purkinje cells recorded during eupneic respiration. These cells had a median complex spike firing frequency of 1.37 Hz (IQR: 0.54 Hz) and a median simple spike frequency of 64.5 (IQR: 34.9) Hz. **E** Violin plots indicating the distributions of the maximal (absolute) complex spike and simple spike modulation for each Purkinje cell during the respiratory cycle. The firing rate modulation is expressed as Z score related to the bootstrap analysis. Responses exceeding a Z score of 3 ($p < 0.01$) were considered to be statistically significant, but it is clear that most Purkinje cells show at least some degree of modulation and any clear separation between modulating and non-modulating Purkinje cells would be subjective. Gray lines in the violin plots indicate 10th, 25th, 50th, 75th and 90th percentiles. **F** There was no significant correlation between the complex spike rate and the depth of the complex spike modulation during the respiratory cycle ($r = 0.133$, $p = 0.396$, Spearman rank correlation test; left). In line with this, the complex spike firing rates of Purkinje cells with weak ($Z<3$) or strong ($Z>3$) complex spike modulation were similar ($U = 148$, $p = 0.895$, Mann-Whitney test). **G** Heat map of the same complex spike data as represented in Fig. 1B, but now ordered per mouse. The horizontal black lines separate the cells recorded in individual mice. **H** The same as in **G**, but now for the simple spikes.

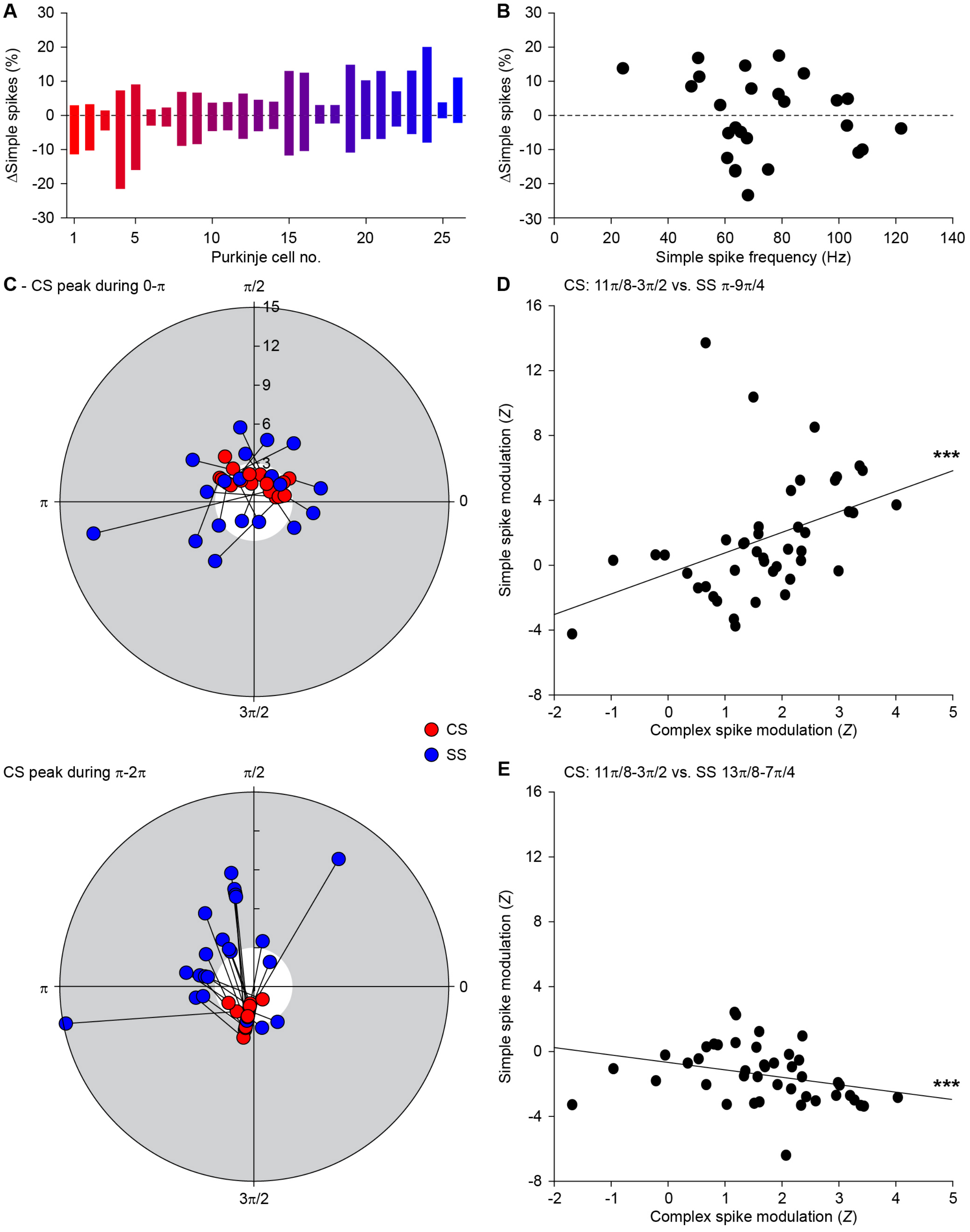
A
ΔSimple spikes (%)
Purkinje cell no.
B
ΔSimple spikes (%)
Simple spike frequency (Hz)
C - CS peak during 0-π
π/2
π
0
3π/2
CS
SS
CS peak during π-2π
D
CS: 11π/8-3π/2 vs. SS π-9π/4
Simple spike modulation (Z)
Complex spike modulation (Z)
***
E
CS: 11π/8-3π/2 vs. SS 13π/8-7π/4
Simple spike modulation (Z)
Complex spike modulation (Z)
***

Figure S2 – Complex spike and simple spike modulation occur during distinct phases of the respiratory cycle, Related to Fig. 2

A Maximal increase and decrease in simple spike firing in response to air puff stimulation per Purkinje cell. The cells are sorted based upon their bias towards decreased (left) of increased (right) simple spike firing. The Purkinje cells have the same color code as in Fig. 2C. **B** No correlation between average simple spike firing rate and maximal simple spike rate modulation (r = -0.14, p = 0.487, Spearman rank correlation test). **C** Polar plot showing, for each Purkinje cell, the relation between the phase of maximal complex spike (red) and that of the strongest simple spike (blue) modulation during unperturbed respiration. The radial axis indicates the modulation strength (in absolute Z score). The grey area indicates $|Z| > 3$. The neurons are separated based on the occurrence of the peak complex spike modulation during the first (top) or second half (bottom) of the respiratory cycle. **D** There was a positive correlation between the rate of simple spike firing around the transition between inspiration and post-inspiration ($\sim\pi$) and the occurrences of complex spikes during the transition from post-inspiration to expiration ($\sim 3\pi/2$) (r = 0.54, $p < 0.001$, Spearman rank correlation). **E** Likewise, there was a negative correlation between complex spike firing around the transition from post-inspiration to expiration and the simple spike rate during expiration ($\sim 7\pi/4$) (r = -0.43, p = 0.004, Spearman rank correlation).

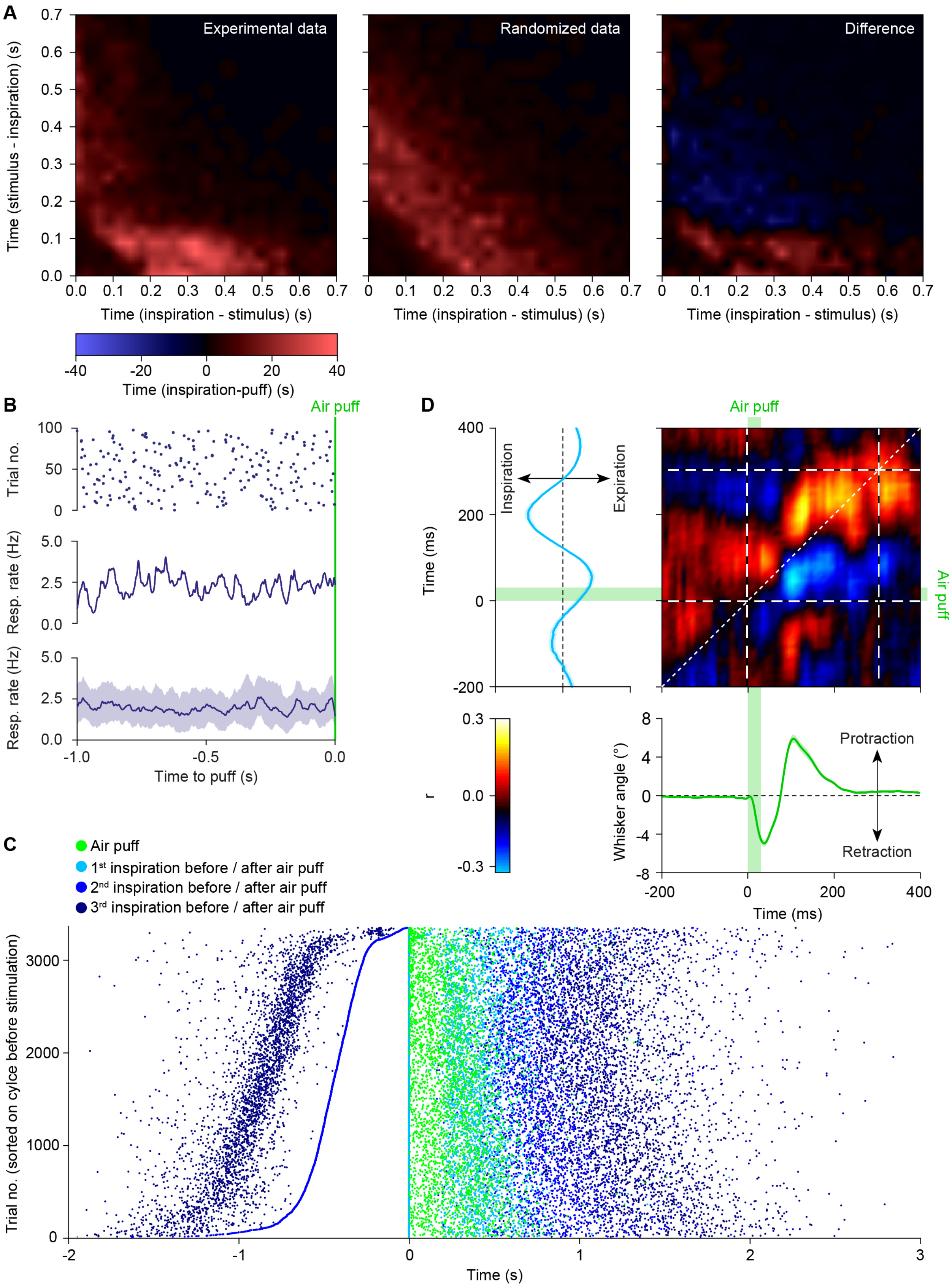

A
Experimental data
Randomized data
Difference
Time (stimulus - inspiration) (s)
Time (inspiration - stimulus) (s)
Time (inspiration-puff) (s)
B
Air puff
Trial no.
Resp. rate (Hz)
Resp. rate (Hz)
Time to puff (s)
C
Air puff
1st inspiration before / after air puff
2nd inspiration before / after air puff
3rd inspiration before / after air puff
Trial no. (sorted on cylce before stimulation)
Time (s)
D
Air puff
Inspiration
Expiration
Time (ms)
Air puff
r
Whisker angle (°)
Protraction
Retraction
Time (ms)

Figure S3 - Whisker pad stimulation triggers inspiration, Related to Fig. 3
A Heat maps showing the distributions of the intervals between the start of the preceding inspiration and the whisker pad stimulation (x axis) and the intervals between whisker pad stimulation and the start of the next inspiration (y axis). The recorded data were compared to data in which the times of the inspiration were randomly shuffled (cf. Fig. 3E-F). In the randomized data (middle), there is a clear symmetry between the time interval between the onset of inspiration and that of the and the time interval from stimulus onset to the start of the next inspiration. This symmetry is broken in the experimental data (left), showing a tendency to start the next inspiration within 100 ms of the stimulus (right). **B** Raster plot of the onset of inspiration (top) and instantaneous respiratory frequency (middle) of an exemplary mouse, showing the breathing rate in the second prior to air puff stimulation. These data, as well as the median instantaneous firing rate of 12 mice (bottom), illustrate the lack of systematic entrainment of inspiration to the air puff. Shaded area indicates inter-quartile range. **C** Whisker pad air puff stimulation affected the timing of the subsequent inhalations, but the mice did not entrain their respiration on the fixed frequency of the air puff stimulation. This becomes clear from the raster plot showing the timing of the start of inspiration around the moment of air puff stimulation. The raster plot is constructed by combining trials from 12 mice, sorted on the duration of the cycle prior to the air puff stimulation. **D** Air puff stimulation of the whisker pad induces a reflexive protraction of the facial whiskers that follows an initial, largely passive backwards movement (green trace, bottom). The same sensory stimulus also accelerates inspiration (cyan trace, left). Trial-by-trial variance analysis indicates that the execution of both behaviors is correlated: whisker protraction is linked with a delay to inspiration. The heat map and the traces are the averages of the 11 mice for which whisker data, of 100 trials per mouse, were available (shaded areas: SEM).

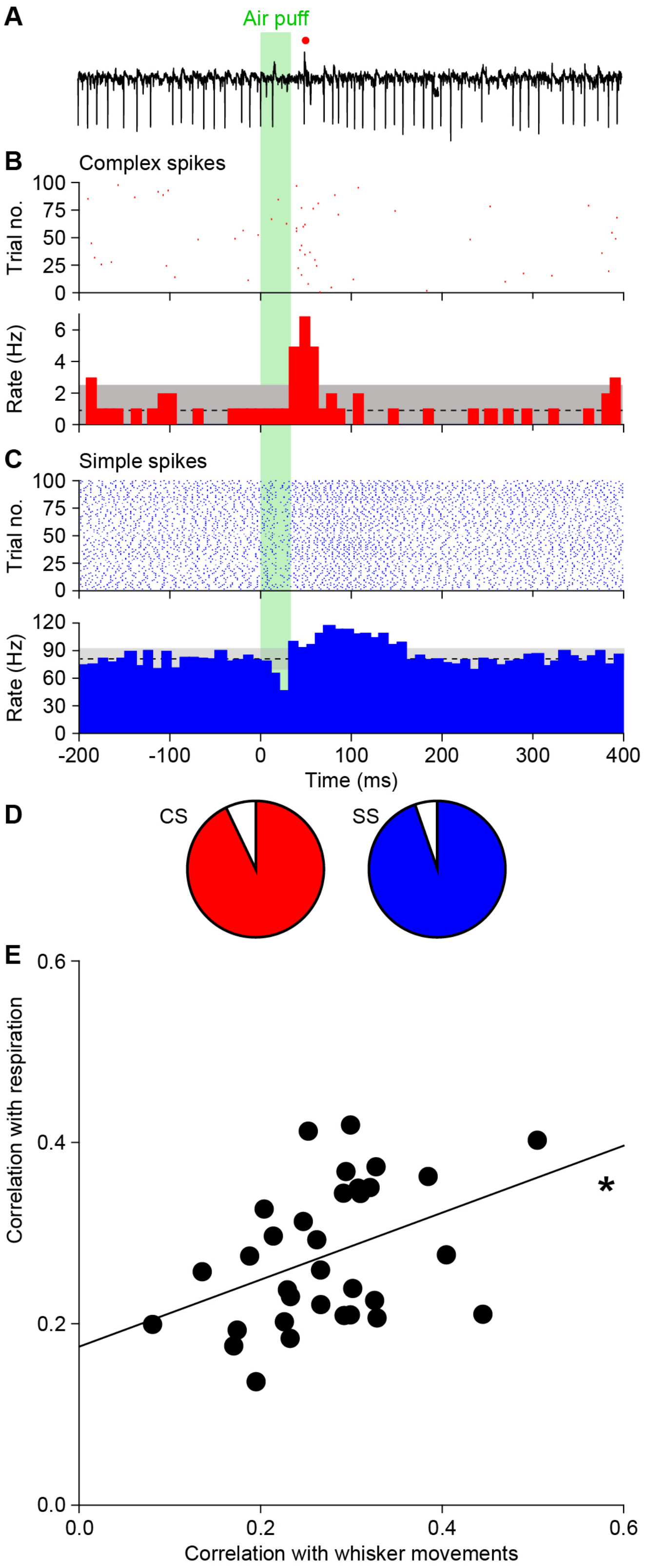

Figure S4 – Whisker pad stimulation triggers Purkinje cell responses, Related to Figs. 4 and 5
A Extracellular recording of a representative Purkinje cell in crus 1 during air puff stimulation of the ipsilateral whisker pad. Of this same cell, raster plots and peri-stimulus time histograms of the complex spikes (**B**) and simple spikes (**C**) were made. Note the bidirectional modulation of the simple spikes. **D** Of the 57 recorded Purkinje cells, 53 (93%) responded with a statistically significant complex spike response to the whisker pad air puff. For the simple spikes, this number was 54 (95%). **E** Based upon the trial-by-trial variations, we calculated the maximal correlation between fluctuations in simple spike frequency and those in whisker position (x axis) and that between fluctuations in simple spike frequency and in inspiration (y axis). The maximal correlations were taken along the 45° line (see Fig. 5C-D). There appeared to be a positive correlation between these two correlations (r = 0.44, $p = 0.010$, Spearman rank correlation), implying that the stronger Purkinje cell simple spike activity was correlated with whisker movement, the stronger the correlation of simple spikes from the same Purkinje with inspiration.

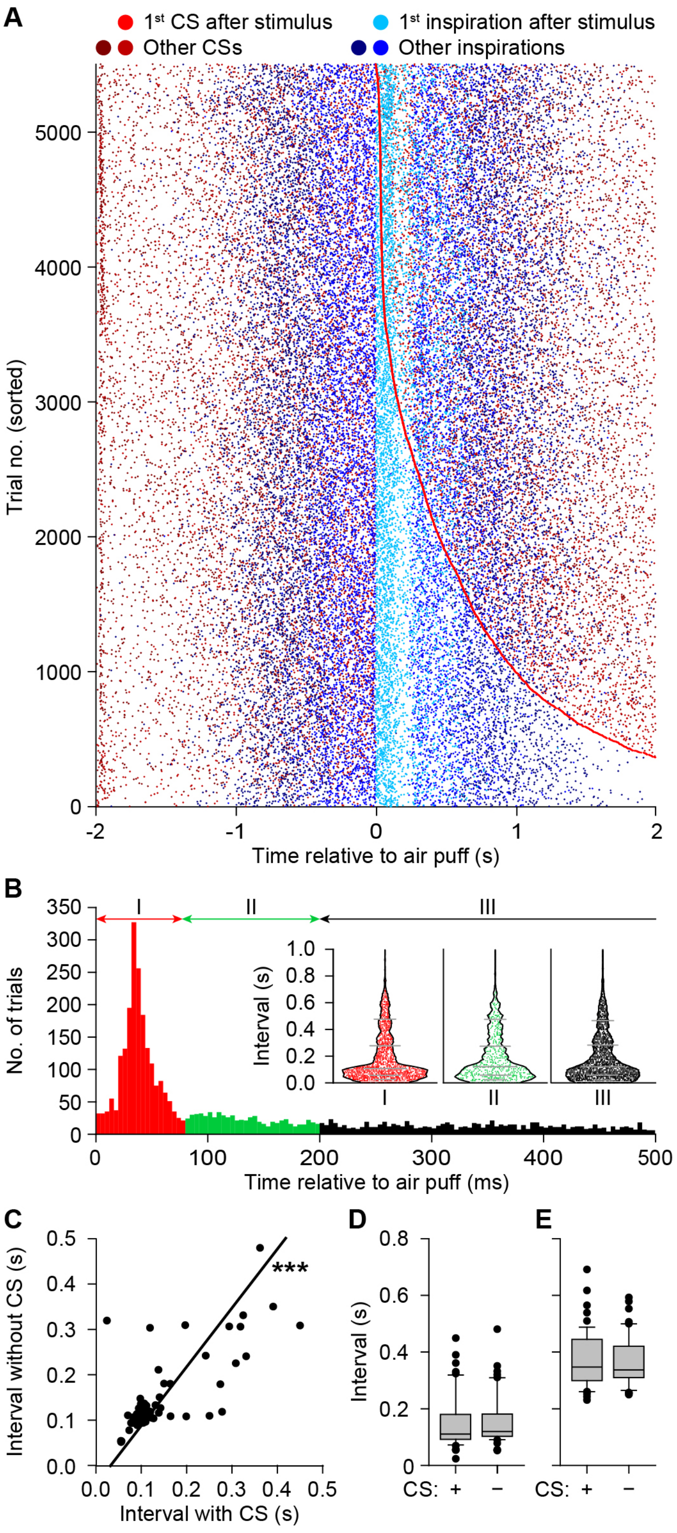

Figure S5 - Complex spikes do not mediate the accelerated inspiration after whisker pad stimulation, Related to Fig. 4

A Air puff stimulation of the whisker pad triggers both complex spike firing (red dots) and accelerated inspiration (cyan dots). This raster plot shows the pooled trials of the 12 mice ordered on the interval between the start of the stimulus and the first complex spike afterwards. **B** Histogram of complex spikes during the first 500 ms after the air puff, composed of the data shown in **A**. The initial peak response occurs within 78 ms. Inset: Violin plots showing that the timing of the first inspiration after the air puff is not depending on the moment of complex spike firing. Left: trials with a complex spike between 0 and 78 ms after the air puff; middle: 78-200 ms; right: 200-500 ms. $p = 0.560$, KW = 1.158, Kruskal-Wallis test. **C** Scatter plot showing, for each Purkinje cell, the median interval between air puff and start of the next inspiration for trials with and without a complex spike within 78 ms of the air puff. The strong correlation demonstrates a lack of impact of complex spike firing on the start of the next inspiration (r = 0.693, $p < 0.001$, Spearman rank correlation). **D** Box plots of the intervals between the air puff and the start of the next inspiration in trials with and without a complex spike during the first 78 ms after the air puff ($p = 0.148$, Mann-Whitney test). **E** The same for the second respiratory interval after the air puff ($p = 0.302$, Mann-Whitney test).

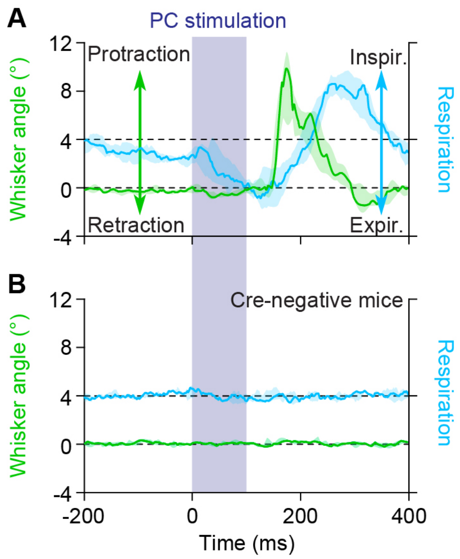

Figure S6 – Purkinje cell stimulation alters both respiratory timing and whisker movements, Related to Fig. 6
A The correlation between Purkinje cell activity, whisker movements and respiration was further tested using optogenetic stimulation of mice expressing channelrhodopsin (ChR2) exclusively in their Purkinje cells (*Pcp2-Ai27* mice). Time course of whisker movements (green) and respiration (blue) recorded simultaneously upon optogenetic stimulation of Purkinje cells in mice expressing ChR2 exclusively in their Purkinje cells (n = 13). **B** No impact of light stimulation in Cre-negative mice that do not express the ChR2 protein (n = 5).

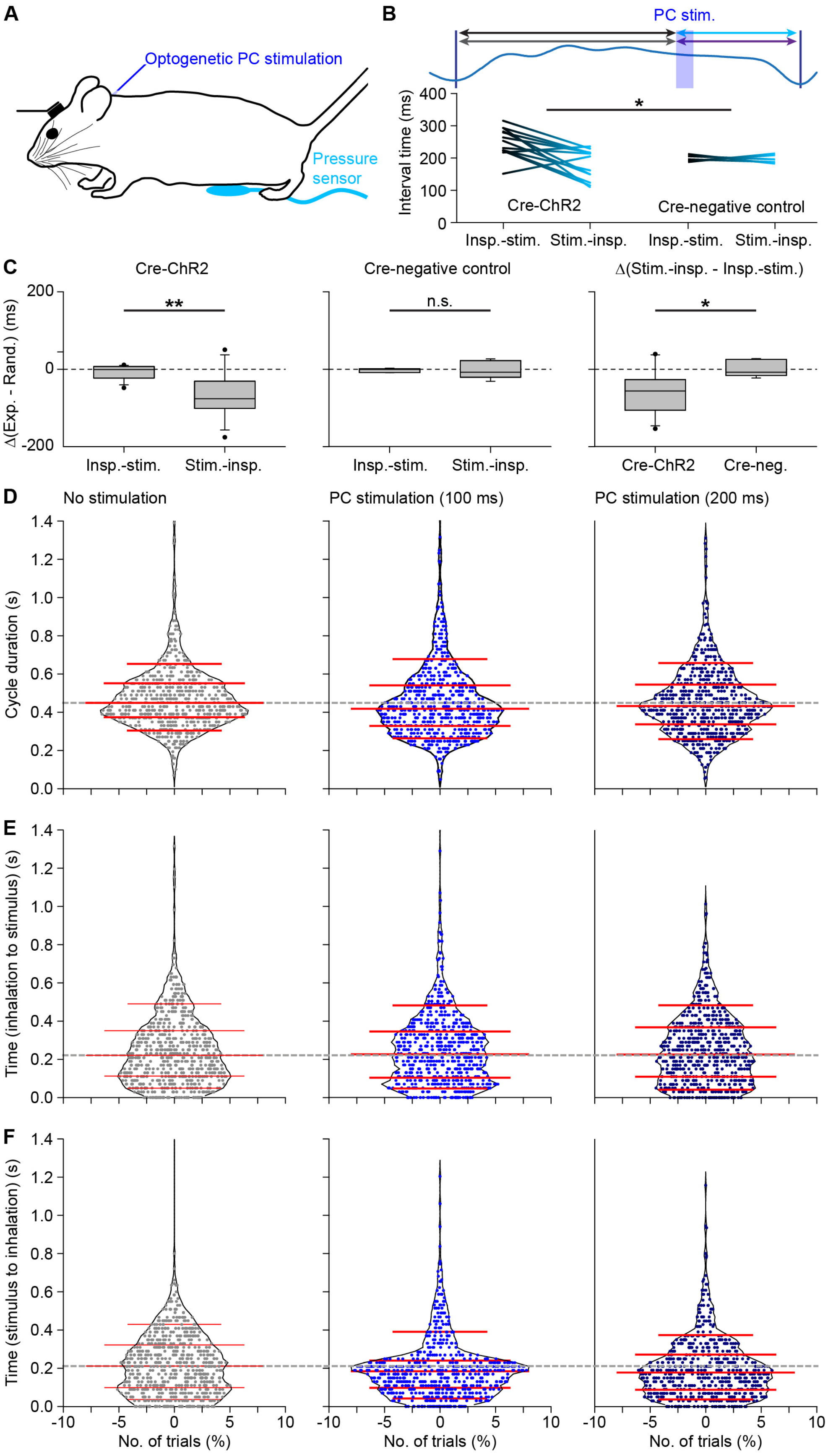
A
Optogenetic PC stimulation
Pressure sensor
B
PC stim.
Interval time (ms)
*
Cre-ChR2
Cre-negative control
Insp.-stim.
Stim.-insp.
Insp.-stim.
Stim.-insp.
C
Cre-ChR2
Cre-negative control
Δ(Stim.-insp. - Insp.-stim.)
Δ(Exp. - Rand.) (ms)
**
n.s.
*
Insp.-stim.
Stim.-insp.
Insp.-stim.
Stim.-insp.
Cre-ChR2
Cre-neg.
D
No stimulation
PC stimulation (100 ms)
PC stimulation (200 ms)
Cycle duration (s)
E
Time (inhalation to stimulus) (s)
F
Time (stimulus to inhalation) (s)
No. of trials (%)
No. of trials (%)
No. of trials (%)

Figure S7 - Purkinje cell stimulation affects respiratory timing, Related to Fig. 6

A The impact of optogenetic stimulation on respiratory timing was studied using transgenic mice expressing ChR2 exclusively in their Purkinje cells and control mice (Cre-negative mice not expressing ChR2 protein). **B** The impact of the light stimulation was different between the ChR2 and control mice (p = 0.030, df = 1, F = 5.686, interaction effect, two-way ANOVA). **C** In comparison to randomized data, optogenetic Purkinje cell stimulation affected the interval between stimulus and inspiration rather than that between the preceding inspiration and the stimulus (left, p = 0.006, df = 1, F = 8.423, interaction effect, two-way ANOVA on power transformed data, n = 13 mice). This, however, was not the case in Cre-negative control mice (middle, p = 0.793, df = 1, F = 0.071, interaction effect, two-way ANOVA on power transformed data, n = 5 mice). The differences between optogenetic and Cre-negative control mice were also significant (right, p = 0.045, df = 1, F = 4.361, interaction effect, two-way ANOVA). Violin plots showing the duration of the respiratory cycle during which the stimulus was given (**D**), the interval between the start of inspiration to that of the stimulus (**E**) and the interval between the start of stimulation and that of the next inspiration (**F**). Left column: 100 ms stimulation, right column: 200 ms stimulation. The horizontal lines indicate the 10th, 25th, 50th, 75th and 90th percentiles.
